# Gut microbiota and their metabolites ameliorate acute and chronic colitis in mice via modulating Th17/Treg balance

**DOI:** 10.3389/fmicb.2025.1643209

**Published:** 2025-08-12

**Authors:** Dongyue Li, Huiling Tao, Xin Tan, Hao Ling, Yue Lu, Huichao Zhang, Sok Theany, Hongyu Xu

**Affiliations:** Department of Gastroenterology, First Affiliated Hospital of Harbin Medical University, Harbin, Heilongjiang, China

**Keywords:** ulcerative colitis, butyrate, Th17, Treg, gut microbiota, chronic phase

## Abstract

**Introduction:**

Ulcerative colitis (UC) is a recurrent inflammatory bowel disease affecting the colorectum, which remains a prominent research focus due to significant individual variations in clinical therapeutic outcomes. Fecal microbiota transplantation (FMT), as a therapeutic approach to restore intestinal homeostasis, has demonstrated favorable efficacy in UC management. However, given the characteristic alternating cycles of active and remission phases in UC, there remains a paucity of in-depth research regarding the optimal timing for FMT intervention. Concurrently, butyrate - a crucial microbial metabolite - ameliorates murine colitis through both direct and indirect mechanisms, while the therapeutic effectiveness of FMT in UC correlates closely with intestinal butyrate concentration.

**Methods:**

This study established acute and chronic UC murine models and employed FMT and butyrate interventions to monitor dynamic alterations in gut microbiota and lymphocyte subsets. Through comprehensive analyses, we aimed to elucidate the interplay between gut microbiota and host immune mechanisms, identify the optimal therapeutic timing for UC interventions, and evaluate the mechanistic role of butyrate. These findings provide theoretical foundations for personalized microbiota-targeted therapies in UC.

**Results:**

Our findings demonstrate that gut microbiota and their metabolites exert therapeutic effects on murine acute/chronic colitis through modulation of the T helper cell 17 (Th17)/T regulatory cell (Treg) ratio. Specifically, the remission phase represents a more favorable window for intestinal homeostasis modulation, with combination therapy involving microbial metabolites exhibiting superior anti-inflammatory efficacy.

**Discussion:**

The maintenance of an appropriate Th17/Treg equilibrium during microbiota restoration demonstrates therapeutic advantages. Notably, butyrate synergistically enhances microbial therapeutic effects, providing experimental evidence for personalized modulation of gut ecosystems in inflammatory bowel disease management.

## 1 Introduction

In recent years, the advancing insights into gut ecosystems have gradually garnered attention for their therapeutic potential in Ulcerative colitis (UC). While Fecal microbiota transplantation (FMT) has demonstrated moderate efficacy in UC management (Paramsothy et al., 2017), marked interindividual heterogeneity persists and its underlying mechanisms remain not yet fully elucidated. Current FMT research in UC has predominantly focused on therapeutic outcomes and safety profiles, whereas critical aspects including the identification of optimal intervention timing across UC disease progression and mechanistic investigations into microbial metabolites (e.g., butyrate) remain underexplored. Notably, delineating the therapeutic window for FMT and elucidating the functional mechanisms of microbial-derived metabolites are imperative for enhancing treatment efficacy and advancing precision therapeutic strategies.

Consistent with clinical observations, histopathological analysis revealed distinct phase-specific characteristics in UC. During the acute phase, H&E staining exhibited colonic ulceration, mucosal edema, goblet cell depletion, crypt distortion with abscess formation, and variably graded inflammatory cell infiltration within the mucosa and submucosa, accompanied by epithelial denudation. In contrast, chronic phase specimens demonstrated attenuated mucosal edema and ulceration, with histological evidence of epithelial hyperplasia, mucosal fibrosis, lymphadenopathy, persistent inflammatory infiltration, and occasional granulomatous changes or tumor-like architectural remodeling. These findings collectively indicate pathohistological disparities between acute and chronic UC phases, suggesting phase-dependent differential immune responses to gut microbiota. Notably, current FMT research predominantly focuses on mild-to-moderate UC cases, while therapeutic exploration in severe UC and stable-phase management remains substantially underexplored.

The elevated T helper cell 17 (Th17) /T regulatory cell (Treg) ratio in UC patients constitutes a pivotal immunopathogenic mechanism (Geremia et al., 2014; Gong et al., 2016). Previous studies have established that Th17 cells characteristically secrete Interleukin17 (IL-17), predominantly exerting pro-inflammatory effects in the intestinal milieu (Yamada et al., 2016). In contrast, Treg cells produce Transforming growth factor-β1 (TGF-β1) and Interleukin10 (IL-10) to suppress effector T-cell activation, thereby maintaining immune tolerance and regulatory functions (Noack and Miossec, 2014). Intestinal Tregs critically inhibit inflammation triggered by dietary antigens or microbial stimuli while preserving epithelial barrier integrity (Izcue et al., 2009). Notably, refractory UC patients demonstrate significant clinical, endoscopic, and histologic remission following adoptive autologous Treg transfer (Voskens et al., 2023). Mechanistically, Treg-mediated suppression encompasses proliferation inhibition of multiple effector T cells including Th17 populations (Ueno et al., 2018). However, UC pathogenesis involves insufficient Treg expansion coupled with Th17 overactivation, creating Th17/Treg imbalance that perpetuates colonic inflammation (Geremia et al., 2014).

Our experimental data revealed acute-phase predominance of Th17 cells with relative Treg depletion (*p* < 0.05 vs. chronic phase), resulting in significant Th17/Treg dysregulation. Intriguingly, chronic-phase specimens exhibited partial Treg reconstitution and substantially normalized Th17/Treg ratios (*p* < 0.05). These findings suggest that dynamic Th17/Treg rebalancing during chronic colitis facilitates intestinal immune homeostasis through mucosal immunomodulation. Consequently, this investigation systematically evaluates Th17/Treg ratio fluctuations across disease phases, aiming to identify optimal FMT intervention windows for UC management.

Short-chain fatty acids (SCFAs), the most extensively studied anti-inflammatory microbial metabolites including acetate, butyrate, and propionate, are generated through anaerobic fermentation of insoluble dietary fibers by gut microbiota. Serving as the primary energy source for colonocytes, they exert trophic effects on intestinal mucosa (Salvi and Cowles, 2021). Pioneering studies have revealed SCFAs’ capacity to epigenetically modulate Th17/Treg cell plasticity, thereby orchestrating immune homeostasis (Ohnmacht et al., 2015; Smith et al., 2013). Specifically, butyrate rebalances immune cell metabolism by dual mechanisms: (1) Histone deacetylase 3 (HDAC3) inhibition-mediated *c-Myc* downregulation suppresses glycolysis - the dominant energy pathway for pro-inflammatory Th17 cells (Zhang et al., 2019); (2) Peroxisome proliferator activated receptor *γ* (PPARγ) activation promotes metabolic reprogramming toward oxidative phosphorylation, the preferential energy metabolism of Tregs (Wang et al., 2016).

Our data demonstrated that dextran sulfate sodium (DSS) challenge activated the Nuclear factor kappa-B (NF-κB) /Interleukin6 (IL-6) /Signal transducer and activator of transcription 3 (STAT3) axis, which was potently reversed by butyrate intervention. Mechanistically, butyrate inhibits NF-κB signaling through stabilizing IκBα (via suppressed proteasomal degradation), blocking nuclear translocation, and impairing DNA binding capacity of NF-κB (Kelly et al., 2004). This pathway is primarily activated in colonic epithelial cells and lamina propria immune cells (e.g., macrophages and dendritic cells), which secrete IL-6 upon DSS-induced damage, subsequently promoting STAT3 phosphorylation in CD4 + T cells to drive Th17 differentiation. Crucially, this pathway intricately regulates Th17/Treg equilibrium: IL-6 synergizes with TGF-*β* to activate STAT3, driving Th17 differentiation via Retineic-acid-receptor-related orphan nuclear receptor *γ* (RORγt) upregulation (Harbour et al., 2020; Durant et al., 2010), while simultaneously suppressing TGF-β-induced Forkhead box protein P3 + (FOXP3+) Treg generation (Bettelli et al., 2006). Pathological activation of this cascade induces profound Th17/Treg imbalance, perpetuating colonic inflammation (Liang et al., 2013), whereas its inhibition restores immune equilibrium (Zhao et al., 2021).

This investigation systematically compares the therapeutic efficacy of butyrate versus FMT, elucidates butyrate’s mechanistic suppression of DSS-induced NF-κB/IL-6/STAT3 hyperactivation, and deciphers its immunometabolic regulation of Th17/Treg dynamics. Complementary *in vitro* evidence confirms butyrate’s preferential differentiation of naïve CD4 + T cells from Inflammatory bowel disease (IBD) patients toward Tregs rather than Th17 lineages (Wen et al., 2021) - a shift proven critical for restoring mucosal immune tolerance (Lv et al., 2018; Tong et al., 2021). We thus propose that butyrate-mediated Th17/Treg rebalancing in UC murine models is mechanistically rooted in NF-κB/IL-6/STAT3 pathway inhibition. Furthermore, strategic supplementation of butyrate-producing bacteria or exogenous butyrate during FMT may synergistically enhance UC remission rates.

This study established acute and chronic UC murine models to investigate the immunological mechanisms underlying butyrate and gut microbiota interventions. By employing combined butyrate administration and FMT, we systematically elucidated the mechanistic interplay between microbial metabolites and Th17/Treg equilibrium. Through multidimensional analysis of butyrate-mediated immunomodulation and microbiota dynamics, this work aims to identify phase-specific therapeutic windows and develop personalized microbiota-targeted regimens for optimized UC management.

## 2 Materials and methods

### 2.1 Reagents

DSS with a molecular weight of 36–50 kDa was purchased from MP Biomedicals. Ltd. Rabbit anti-NF-κB p65, anti-STAT3 polyclonal antibodies were purchased from Jiangsu Meimian industrial Co., Ltd. Fecal occult blood reagent was purchased from Shanghai Wei dysprosium Biotechnology Co., Ltd.

### 2.2 Animal and FMT

All specific pathogen-free (SPF) C57BL/6 mice (6–8 weeks old, 25 ± 2 g) were procured from Liaoning Changsheng Biotechnology Co., Ltd. The experimental protocol was approved by the Animal Ethics Committee of The First Affiliated Hospital of Harbin Medical University (Approval no. 2022122) and strictly adhered to ARRIVE guidelines. Fifty mice were stratified into five experimental groups (*n* = 10/group):blank control group, DSS group, DSS + FMT group, DSS + butyrate group and DSS chronic group. All mice except the blank control group drank 3% DSS (molecular weight 36,000–50,000 Da, MP Biomedicals, CA, USA) solution for 7 days, and all of them were replaced with mineral water after 7 days. Mice in DSS + butyrate group were given 0.5% sodium butyrate solution (Sigma-Aldrich, St. Louis, MO, USA) by gavage every day, while mice in DSS + FMT group were given fecal bacteria filtrate of blank control group by gavage every day. The chronic DSS group drank 2.5%DSS alternately for three cycles (namely, the 1st to 5th, 11th to 15th, 21st to 25th days, and the rest time). For 30 days, the other mice were given normal saline by gavage, and the amount of gavage was 0.2 mL. On the 14th day, the mice were killed, and the colon tissue and spleen of the mice were taken for subsequent analysis. The body weight, fecal occult blood, and water consumption of mice were recorded daily during the experiment. Following 14-day interventions, fecal samples were collected for microbial analysis, colon tissues and splenic lymphocytes were harvested for histopathological/immunological evaluations.

Fresh fecal filtrate was prepared daily: Fresh feces from 5 to 7 healthy control mice (defecation stimulated by gentle abdominal massage) were collected into sterile 15-mL tubes. Five volumes of anaerobic PBS were added, followed by 10-min static incubation under oxygen-free conditions. After thorough vortexing, the suspension was filtered through sterile gauze to remove particulate matter. Recipient mice received 1 μL/10 g body weight of the filtrate via oral gavage.

### 2.3 Disease activity index (DAI) score

Mice were scored according to daily body weight change, fecal characteristics, and bleeding, and the DAI score was obtained by dividing the three total scores by three. The specific scoring criteria are shown in Table 1.

**Table 1:** Disease activity index (DAI) scores.

Weight loss	Stool consistency	Bleeding stool	Score
No weight loss	Normal	Normal	0
Decrease of 1–5%			1
Down 5–10%	Loose	Occult blood positive	2
Down 10–15%			3
Decrease >15%	Watery	Dominant bleeding	4

### 2.4 HE staining

Colonic tissue sections (4-μm-thick) were prepared and subjected to hematoxylin and eosin (H&E) staining following a standard protocol. Histopathological scoring was performed using the criteria outlined in Table 2 to assess colitis severity.

**Table 2:** Pathological histological scoring criteria.

Part	Performance and rating
Mucosal epithelium	Ulcer formation: none (0); mild surface (1); moderate (2); extensive total (3)
Crypt	Mitotic activity: lower 1/3 (0); mild to moderate 1/3 (1); moderate to moderate 1/3 (2); upper 1/3 (3) Neutrophil infiltration
Mucosal lamina propria	Mucus Defects Plasma cell infiltration Neutrophil infiltration Vascular formation Cellulose deposition: none (0); restricted to mucosal layer (1); submucosal layer (2); wall permeability (3)
Submucosa	Neutrophil infiltration Edema

### 2.5 Immunohistochemical (IHC) staining

To evaluate inflammatory factor expression in colonic tissues, IHC staining was performed. 4-μm colon tissue sample underwent deparaffinization, antigen retrieval, and endogenous peroxidase inactivation. After dewaxing, antigen repair and endogenous peroxidase removal, 4 μm colon tissue samples were incubated with NF-κB p65, STAT3 and IL-6 primary antibody (HUABIO, Hangzhou, China), followed by secondary antibody incubation and DAB staining, which were observed under microscope and scored: three high-magnification visual fields were selected and scored according to the staining degree and positive range. The negative criterion is that the staining is light yellow and the number of positive cells is <: 10%. The criterion of weak positive is that the number of positive cells accounts for 10–25% and the staining is yellow; Positive is between weak positive and strong positive; Strong positive means that the number of positive cells is-50%, and the color intensity is brown; In statistical analysis, the negative score is 0; Weak positive score is 1 point; Positive score is 2 points; A strong positive score is 3 points.

### 2.6 Flow cytometry

DNA from colon tissue was extracted, and DNA integrity detection and PCR amplification were carried out. Both ends of DNA were fixed respectively, and DNA clusters were generated by amplification. dNTP and DNA polymerase with fluorescent labels were added, and fluorescence signals were collected during the synthesis process to obtain sequence information, which was analyzed on Majorbio platform. To analyze the proportions of Treg and Th17 cells within splenic CD4 + T lymphocytes by using polyspecific stimulators, spleens were homogenized into single-cell suspensions, and the concentration was adjusted to 1*106/ml. Leukocyte Activation Cocktail (BD Biosciences, CA, USA) was added to each ml of single cell suspension, and it was stimulated at 37°C and 5% CO_2_ for 6 h. Fixable Viability Stain 780 (BD Biosciences) was incubated for 30 min at room temperature in the dark, so as to distinguish between cell life and death. Labeling cell surface antibody, adding CD4 antibody labeled by FITC and CD25 antibody labeled by PE (BD Biosciences), and incubating at room temperature for 30 min. Add fixed membrane-breaking solution (BD Biosciences) and incubate in the dark for 50 min at 4°C, then stain the cells, add IL-17 antibody labeled with PE CF594 and FOXP3 antibody labeled with Alexa Fluor 647, incubate in the dark for 50 min at 4°C, and immediately detect on the computer after staining.

### 2.7 16S rRNA sequencing

DNA from colon tissue was extracted, and DNA integrity detection and PCR amplification were carried out. Both ends of DNA were fixed respectively, and DNA clusters were generated by amplification. dNTP and DNA polymerase with fluorescent labels were added, and fluorescence signals were collected during the synthesis process to obtain sequence information, which was analyzed on Majorbio platform.

### 2.8 Fecal sample processing and gas chromatography analysis

Approximately 0.05 g of murine fecal sample was homogenized in 1 mL of pre-chilled PBS on ice for 3 min using a tissue homogenizer. Ultrasound at 4°C for 15 min. Centrifuge at 13000 r/min for 10 min. Take 0.5 mL of supernatant and pass it through 0.45 *μ*m filter membrane for testing. Chromatographic conditions are as follows: N2 is used as carrier gas, chromatographic column: Agilent DB-23 (60 m × 0.25 mm × 0.25 μm), inlet temperature: 270°C, detector temperature: 280°C, H2 flow rate: 50 mL/min, N2 flow rate: 30 mL/min, air flow rate: 500 mL/min, split ratio:. Column box: keep the initial temperature of programmed temperature at 80°C for 4 min, then raise it to 180°C at 10°C/min, keep it for 1 min, flow rate: 2 mL/min, and sample volume: 2 μ l.

### 2.9 Statistical analyses

Statistical analyses were performed using SPSS 25.0 (IBM Corp., Armonk, NY, USA). Continuous variables with normal distribution were expressed as mean ± standard deviation (SD), while non-normally distributed data were presented as median and interquartile range (IQR). For multi-group comparisons meeting normality, independence, and homogeneity of variance assumptions, one-way ANOVA was applied, followed by Fisher’s Least Significant Difference (LSD) *post-hoc* tests for pairwise comparisons and rank sum test is used for the comparison of non-normal distribution data. When *p* < 0.05, the difference is considered to be statistically significant.

## 3 Results

### 3.1 Inflammation and flora characteristics of acute and chronic UC

#### 3.1.1 Species diversity analysis

**Figure 1:**
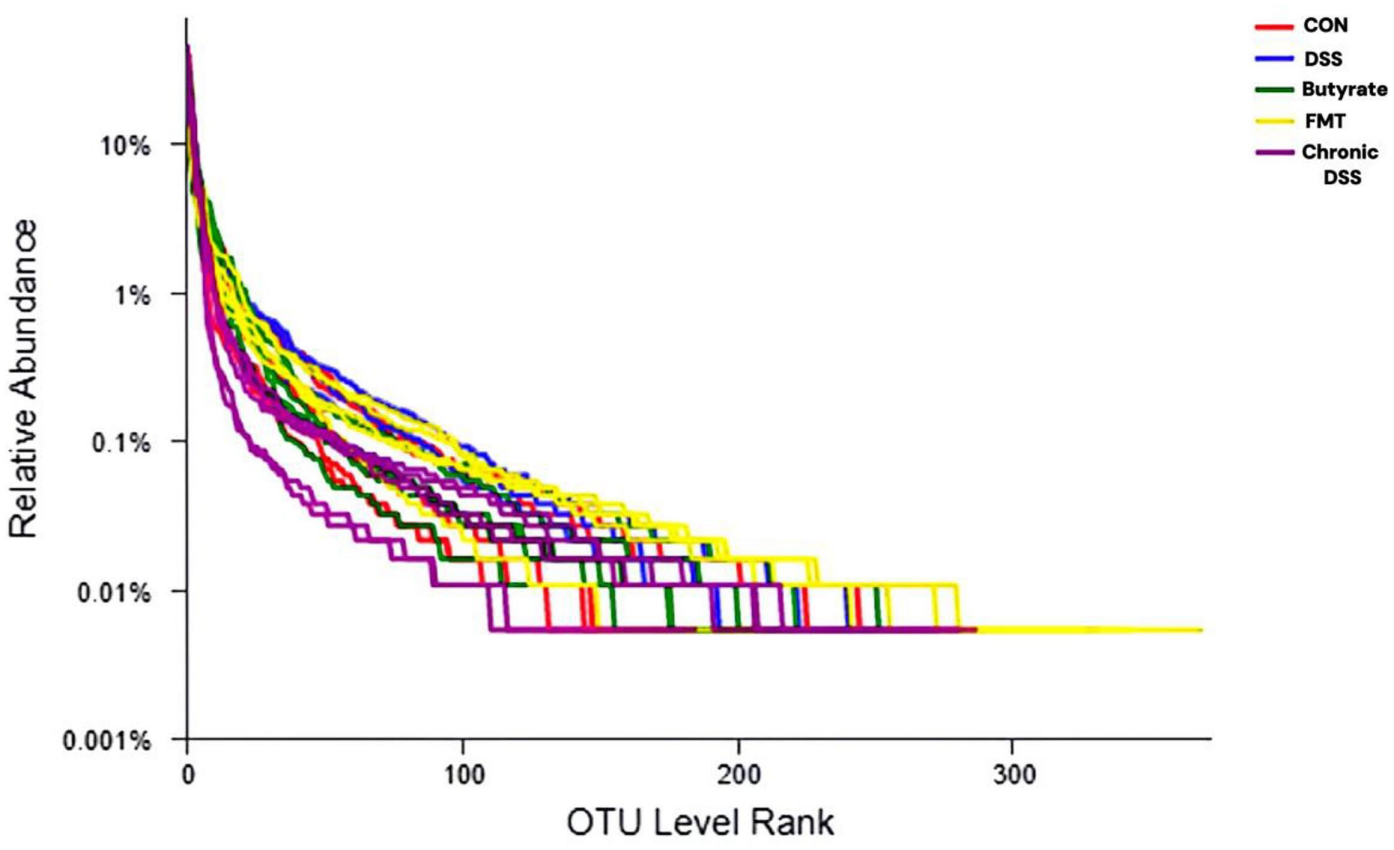
Rank-abundance curve. The x-axis represents the ranked order of species (or OTUs) at a given taxonomic level, while the y-axis indicates the relative percentage abundance of species at that taxonomic level. The terminal point of the curve’s extension on the x-axis corresponds to the species count within the sample. A smooth decline of the curve indicates higher species diversity in the sample, whereas a steep, abrupt decline suggests a predominance of dominant bacterial taxa with reduced diversity.

**Figure 2:**
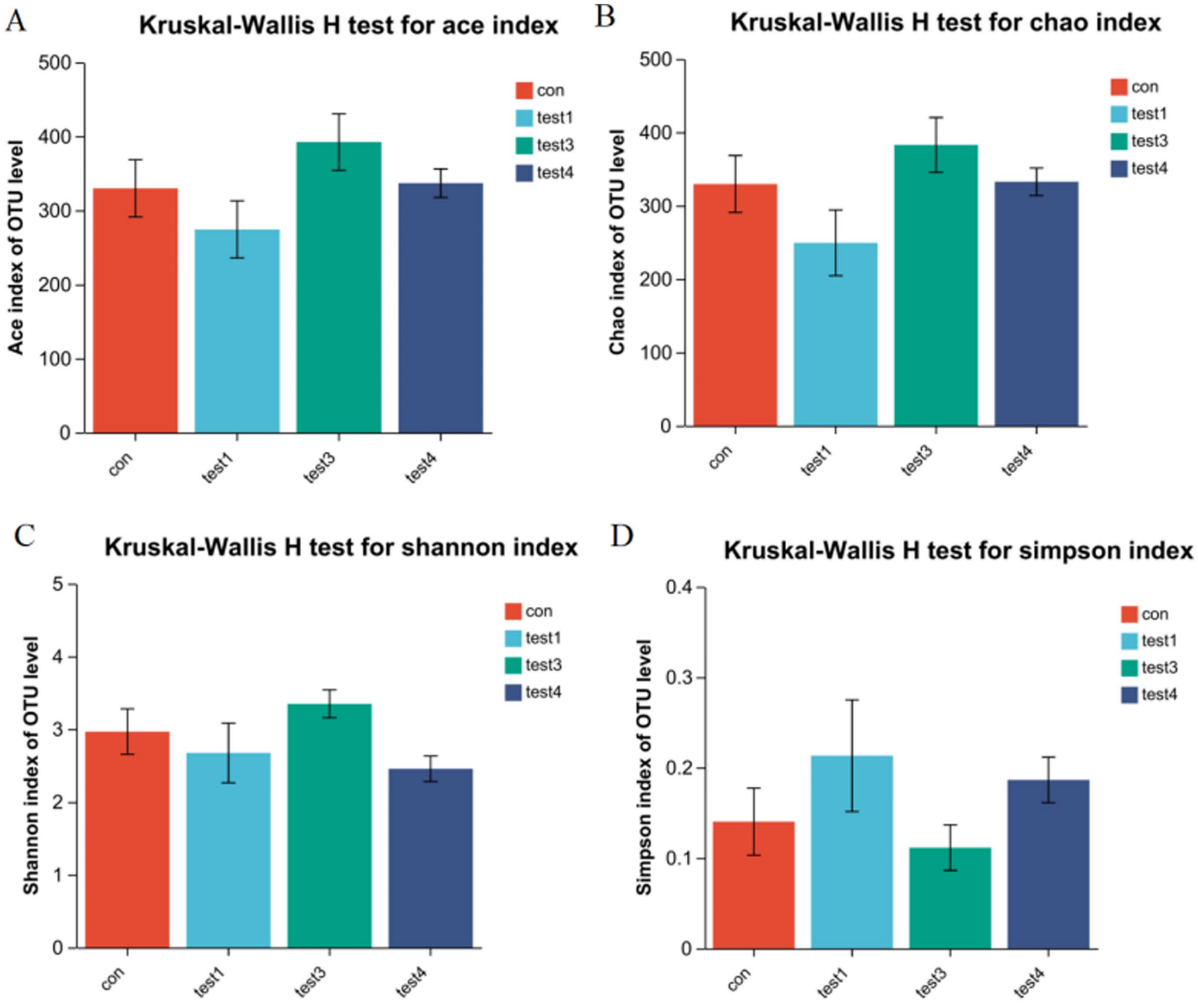
Gut microbiota *α*-diversity analysis. **(A)** ACE index, **(B)** Chao1 index, **(C)** Shannon index, **(D)** Simpson index (*p* < 0.05, ^*^p < 0.01, ^**^p < 0.001). The Chao1 and ACE indices reflect species richness, with higher values indicating greater taxonomic richness. In contrast, the Shannon and Simpson indices characterize microbial diversity. A higher Simpson index combined with a lower Shannon index typically signifies reduced diversity due to dominance by specific taxa (paradoxical interpretation requires validation via complementary metrics).

A Rank-Abundance curve with a slower descent rate and broader extension range indicates higher species diversity. As demonstrated in Figure 1, the chronic group exhibited the lowest species richness, whereas the FMT group displayed the highest species richness. Alpha diversity analysis is shown in Figure 2. The acute group showed significantly decreased Chao1, ACE, and Shannon indices compared to the blank control group (*P* < 0.05), while the Simpson index was markedly increased, indicating reduced richness and diversity of the gut microbiota during acute DSS modeling. In the chronic group, ACE and Chao1 indices were comparable to the control, but Shannon index decreased and Simpson index increased, suggesting a partial decline in microbial diversity. Following FMT intervention, all indices (ACE, Chao1, Shannon, and Simpson) returned to levels similar to the control group, demonstrating that FMT effectively restored microbial richness and diversity.

#### 3.1.2 Microbiota analysis

**Figure 3:**
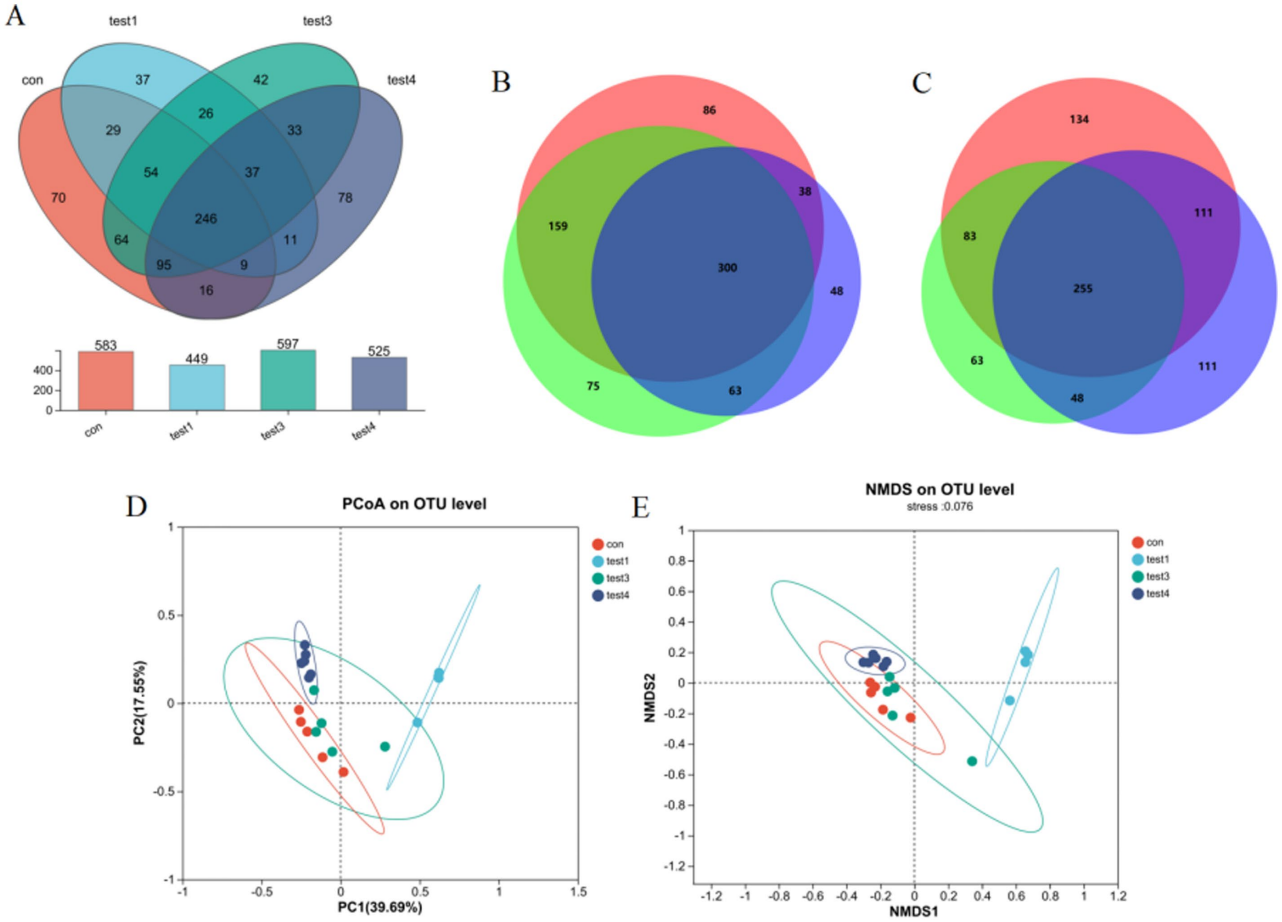
Gut microbiota compositional analysis. **(A)** Four-group Venn diagram, **(B)** Venn diagram comparing acute, FMT, and CON groups, **(C)** Venn diagram comparing acute, chronic, and CON groups, **(D)** *β*-diversity analysis via PCoA, **(E)** NMDS ordination with community composition heatmap.

Microbiota analysis revealed 224 core operational taxonomic units (OTUs) across all five experimental groups. Group-specific OTU distributions were quantified as follows: CON group exhibited 65 unique OTUs (583 total), acute phase group 26 (449), FMT-treated group 29 (597), and chronic phase group 28 (525) (Figure 3A). Venn diagram analysis demonstrated substantial divergence in OTU composition between the acute phase and CON groups, whereas the FMT group showed restored microbial profiles closely resembling the CON group (Figure 3B), indicating enhanced *α*-diversity and microbiota normalization post-FMT intervention. Furthermore, chronic phase microbiota exhibited progressive compositional normalization toward CON group patterns compared to the acute phase cohort (Figure 3C).

#### 3.1.3 *β*-diversity analysis

Principal Coordinates Analysis (PCoA) revealed significant intergroup microbiota divergence, with distinct clustering of the CON group, acute phase cohort, and chronic phase cohort into three separate groups (Figure 3D). Both acute and chronic phase groups exhibited marked compositional deviations from the CON group. Non-metric Multidimensional Scaling (NMDS) analysis further demonstrated a convergence trend of chronic phase microbiota toward CON group clustering (stress value <0.15), suggesting progressive microbial normalization during disease chronicity (Figure 3E).

#### 3.1.4 Genus-level compositional of gut microbiota in acute and chronic colitis models

**Figure 4:**
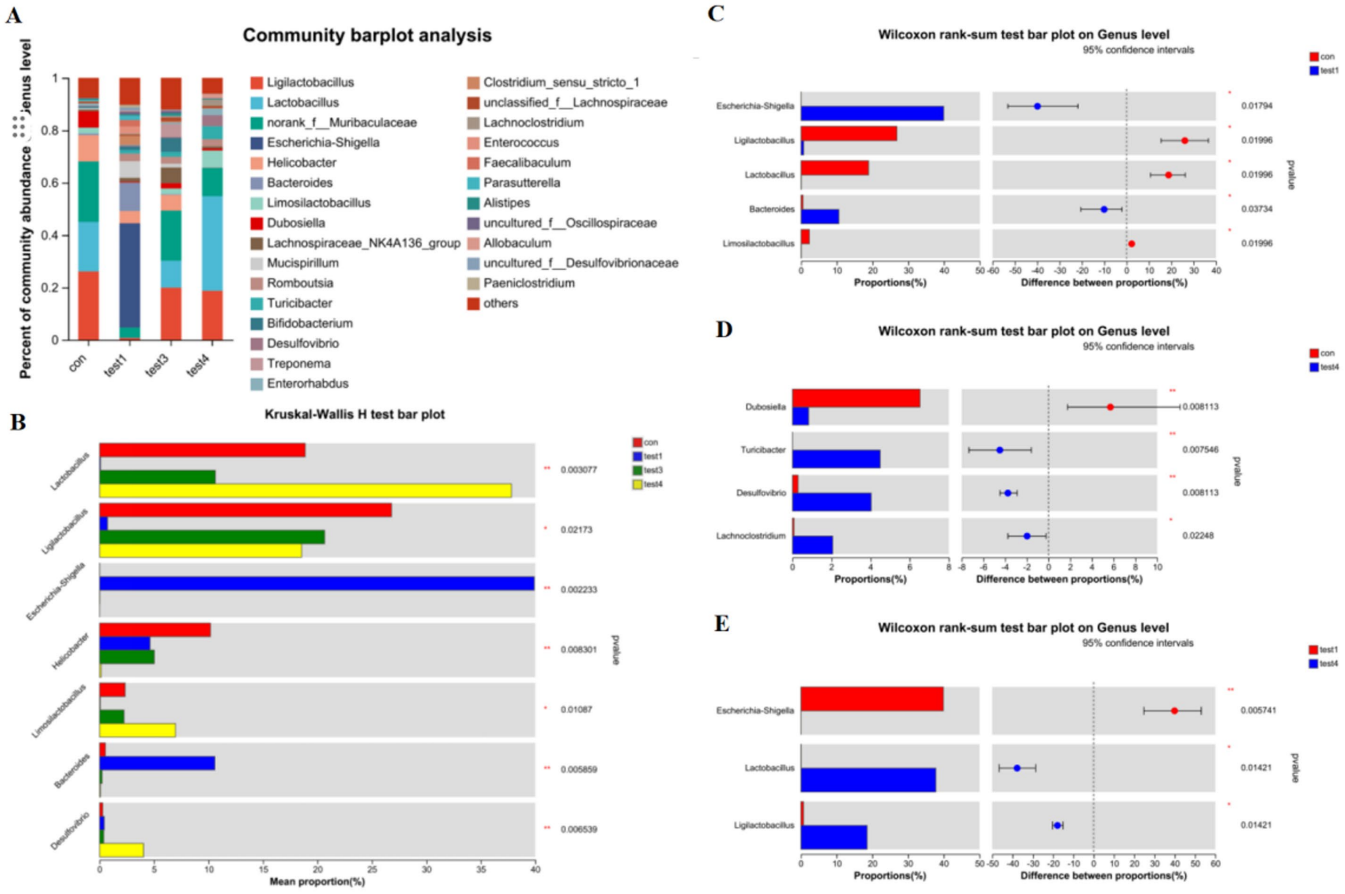
Genus-level taxonomic divergence in gut microbiota of UC mice. **(A)** Taxonomic composition histogram at genus level, **(B)** cross-group differential abundance analysis, **(C)** CON vs. acute group differential testing, **(D)** CON vs. chronic group differential testing, **(E)** acute vs. chronic group differential testing.

Genus-Level Compositional Analysis of Gut Microbiota in Acute and Chronic Colitis Models as shown in Figure 4, acute colitis cohorts exhibited significant microbial dysbiosis at the genus level compared to CON controls. The relative abundances of *Bacteroides* and *Escherichia-Shigella* were markedly increased (*p* < 0.05), while *Ligilactobacillus*, *Lactobacillus*, and *Limosilactobacillus* were substantially depleted (Figures 4A–C).

In contrast, chronic-phase microbiota displayed restoration patterns: *Escherichia-Shigella* enrichment was absent, and *Lactobacillus* abundance rebounded to levels approaching healthy baselines (Figures 4A,B,D). Notably, FMT intervention effectively reconstructed microbial architecture, restoring *Lactobacillus*, *Ligilactobacillus*, and *Limosilactobacillus* to near-healthy relative abundances at the genus level (Figures 4A,E).

#### 3.1.5 Species-level compositional analysis of gut microbiota

**Figure 5:**
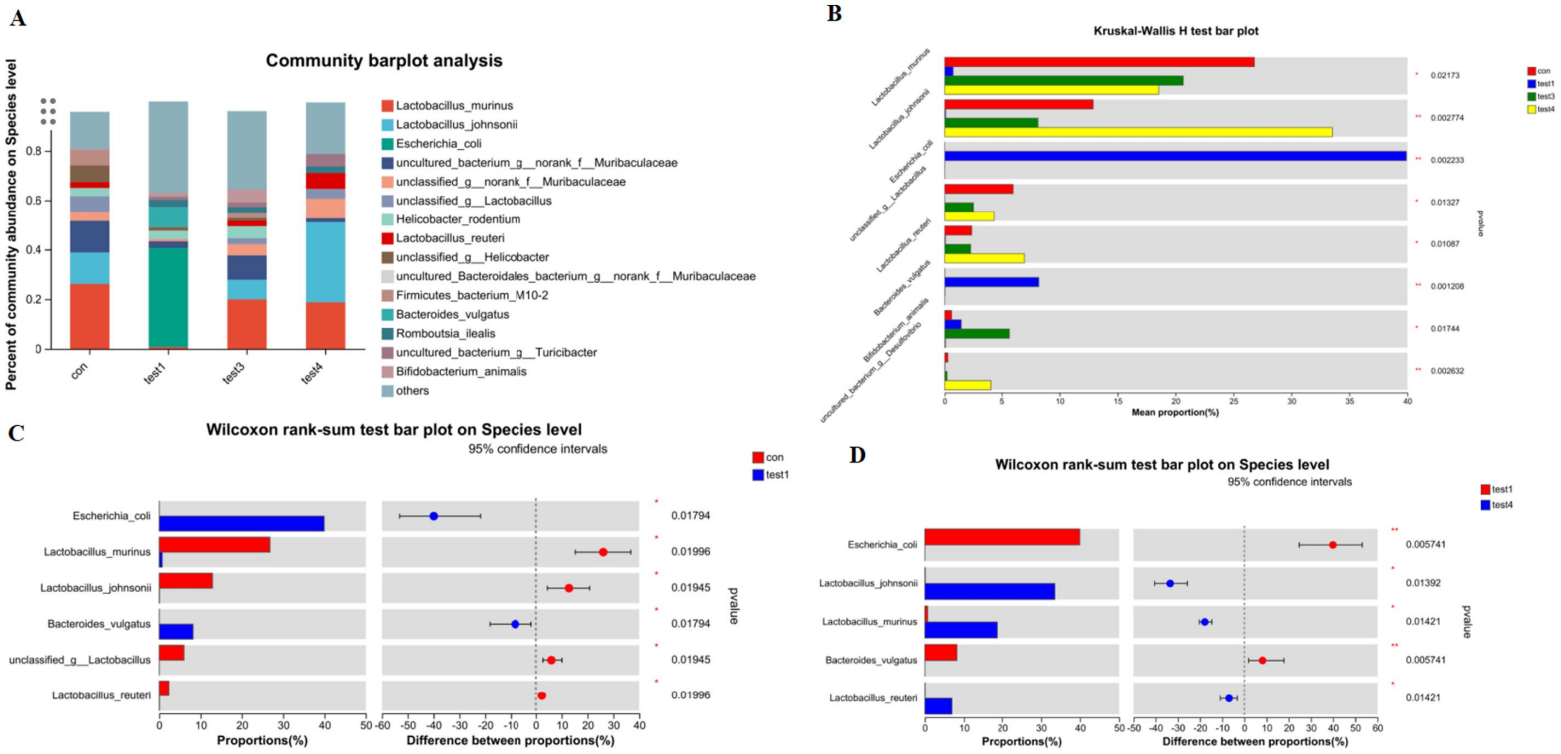
Species-level taxonomic divergence in gut microbiota of UC mice. **(A)** Species composition profiling (top 20 taxa; z-score normalized), **(B)** cross-group differential analysis (Kruskal-Wallis, FDR-adjusted), **(C)** CON vs. acute group comparison (Mann–Whitney U), **(D)** acute vs. chronic group comparison (ANCOM-BC).

Comparative analysis at the species resolution revealed distinct dysbiosis patterns. Acute colitis cohorts exhibited significantly increased relative abundances of potential pathobionts *Escherichia coli* and *Bacteroides vulgatus* compared to CON controls (*p* < 0.05), concurrent with depletion of beneficial *Lactobacillus murinus*, *Lactobacillus johnsonii*, *unclassified Lactobacillus* spp., and *Lactobacillus reuteri* (Figures 5A,B).

Chronic-phase microbiota demonstrated partial restoration, with *Lactobacillus murinus*, *Bifidobacterium animalis*, and *Lactobacillus reuteri* abundances exceeding CON baselines (*p* < 0.05), while *Escherichia coli* showed no significant enrichment (Figure 5D). Notably, chronic-phase specimens exhibited marked recovery of *Lactobacillus johnsonii*, *Lactobacillus murinus*, and *Lactobacillus reuteri* alongside attenuated *Escherichia coli* and *Bacteroides vulgatus* levels relative to acute-phase counterparts (*p* < 0.05).

FMT effectively reconstituted species-level profiles, eliminating pathogenic enrichment and restoring microbiota compositions to near-CON configurations (Figures 5A,B).

### 3.2 Timing of FMT (acute vs chronic UC)

#### 3.2.1 Histopathological features of acute and chronic UC via HE staining

**Figure 6:**
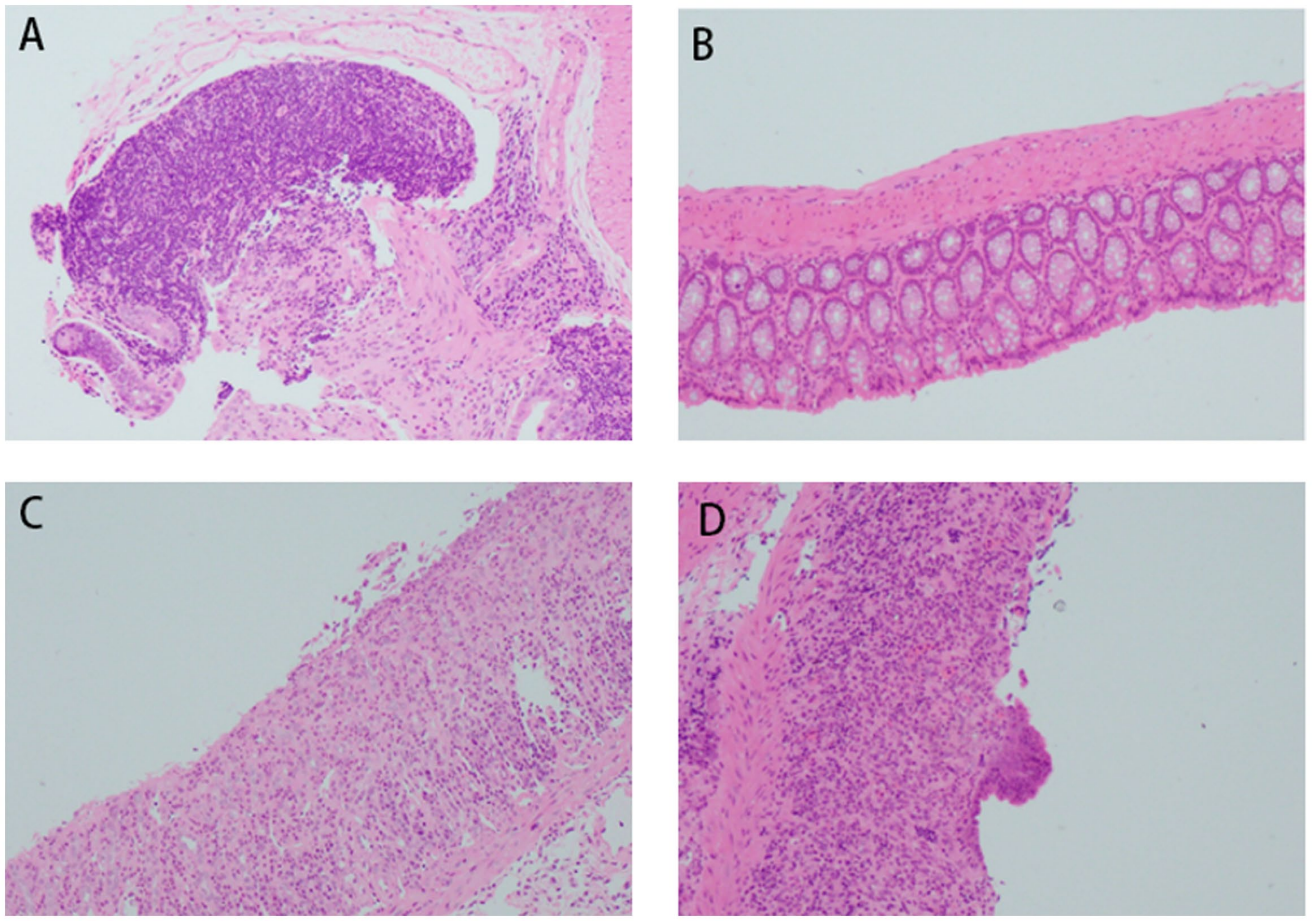
Histopathological changes in mouse colon tissues (H&E staining, ×200 magnification). **(A)** Acute DSS group; **(B)** control group; **(C)** FMT group; **(D)** chronic DSS group.

In the control (CON) group, colonic mucosa exhibited structural integrity without ulceration, edema, or hyperemia. Crypts were tightly arranged with abundant goblet cells, and no significant inflammatory infiltration was observed. The acute UC group displayed colonic ulcers, mucosal edema, goblet cell depletion, crypt distortion, and varying degrees of inflammatory cell infiltration in the mucosa and submucosa, accompanied by epithelial damage and crypt abscess formation in some regions. In contrast, the chronic UC group showed attenuated mucosal edema and ulceration compared to the acute group, alongside epithelial hyperplasia, mucosal fibrosis, lymphoid hyperplasia, and mild-to-moderate inflammatory infiltration. Focal granulomatous changes and tumor-like alterations were occasionally noted. Notably, the FMT group demonstrated reduced colonic inflammation compared to both acute and chronic UC groups, characterized by diminished submucosal inflammatory infiltration and alleviated mucosal edema (Figure 6).

#### 3.2.2 Proportions of Th17 and Treg cells among CD4 + T cells in acute and chronic UC groups

**Figure 7:**
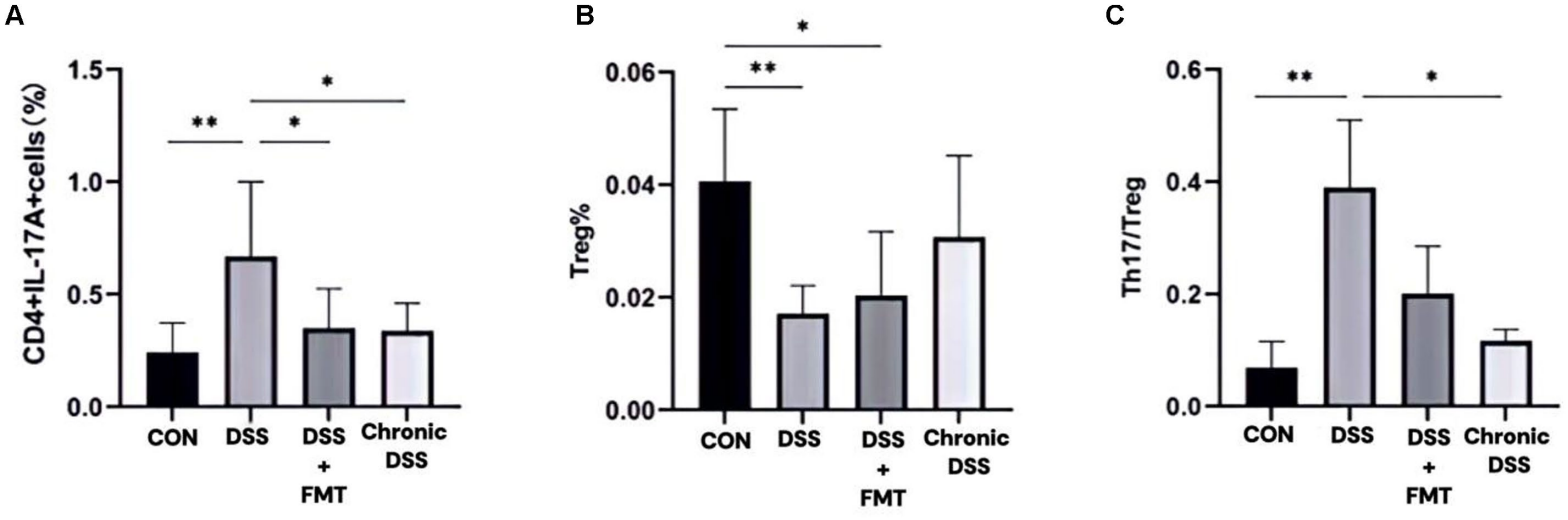
Proportions of Th17 and Treg lymphocytes within CD4^+^T cell populations. **(A)**Th17 cell percentage; **(B)** Treg cell percentage; **(C)** Th17/Treg ratio.

**Figure 8:**
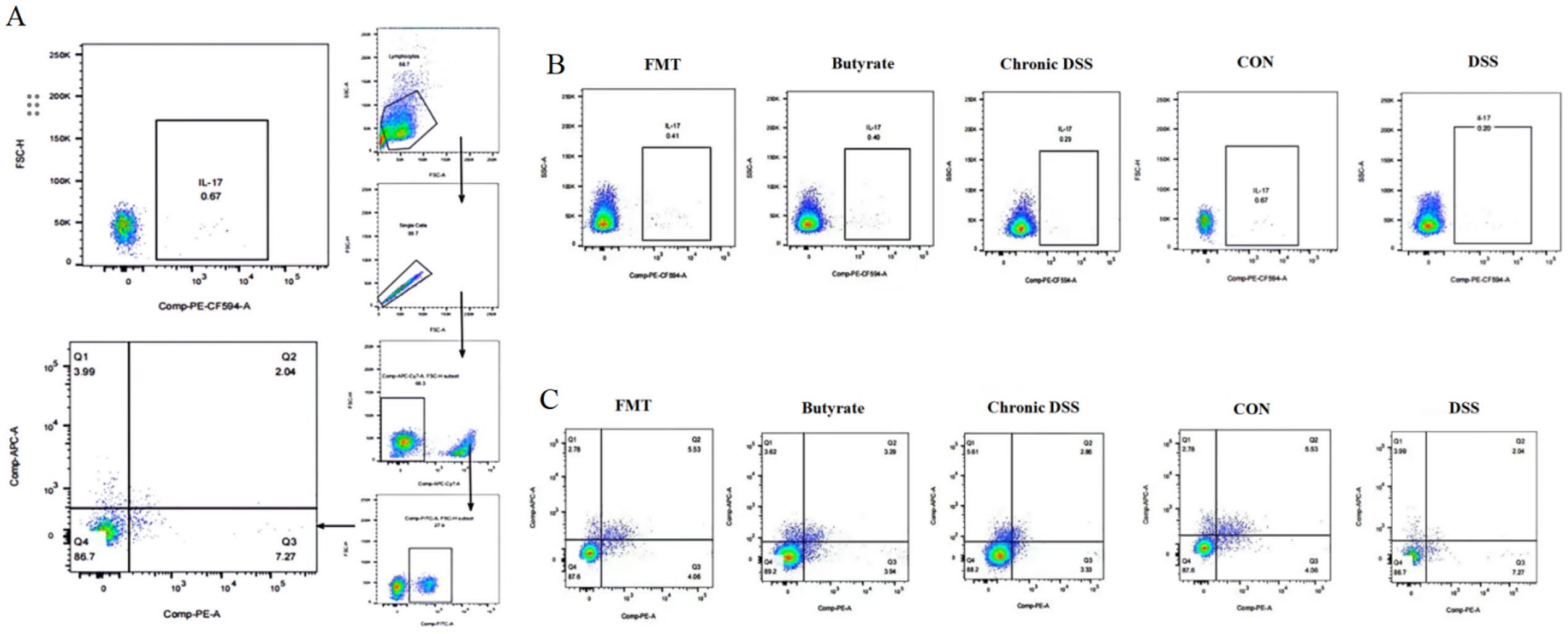
Flow cytometric analysis of splenic single-cell suspensions. **(A)** Gating strategy for Th17 and Treg cells; **(B)** percentage of Th17 cells in CD4^+^T cells; **(C)** percentage of Treg cells in CD4^+^ T cells.

As shown in Table 3, the control group exhibited the lowest proportion of Th17 cells. In contrast, the acute UC group demonstrated a significant increase in Th17 cell frequency compared to all other groups (*p* < 0.05), with no statistically significant differences observed among the remaining groups (Figure 7A). Regarding Treg cell proportions, both the FMT and acute UC groups showed a reduction relative to the control group, reaching statistical significance (Figure 7B). The Th17/Treg ratio was markedly elevated in the acute DSS group, significantly higher than in the control and chronic UC groups (*p* < 0.05) (Figure 7C). The gating strategy for flow cytometry is illustrated in Figure 8A, with representative flow cytometry plots for Th17 and Treg cell proportions across groups shown in Figures 8B,C.

**Table 3:** Treg and Th17 cell frequencies with Th17/Treg ratio across experimental groups.

Group	Th17 (%)	Treg (%)	Th17/Treg
Control	0.25 ± 0.12	4.06 ± 1.28	0.07 ± 0.05
DSS group	0.67 ± 0.33	1.71 ± 0.49	0.39 ± 0.12
DSS + FMT group	0.35 ± 0.17	2.03 ± 1.14	0.20 ± 0.08
Chronic DSS group	0.34 ± 0.12	3.07 ± 1.44	0.12 ± 0.02

#### 3.2.3 Role and mechanism of butyrate in the anti-inflammatory process of UC (possible mechanism of balancing TRG/TH17)

Gas chromatography was employed to quantify fecal butyrate levels in mice subjected to FMT or butyrate intervention. As shown in Table 4, the DSS group exhibited a significant reduction in fecal butyrate content compared to the control group (*p* < 0.001). Conversely, both the FMT and butyrate-treated groups demonstrated markedly increased butyrate levels relative to the DSS group (*p* < 0.01 and *p* < 0.001, respectively).

**Table 4:** Fecal butyrate content across experimental groups.

Group	Butyrate (mg/g)
Control	1.36 ± 0.38
DSS group	0.42 ± 0.01^**^
DSS + Butyrate group	2.01 ± 0.43^* ##^
DSS + FMT group	1.16 ± 0.21^#&^

^*^P < 0.01 vs. Control; ^**^p < 0.001 vs. Control; ^#^*p* < 0.01 vs. DSS group; ^##^*p* < 0.001 vs. DSS group; ^&^*p* < 0.001 vs. DSS + Butyrate group.

#### 3.2.4 Proportions of Treg and Th17 cells in splenic lymphocytes under FMT and butyrate interventions

**Figure 9:**
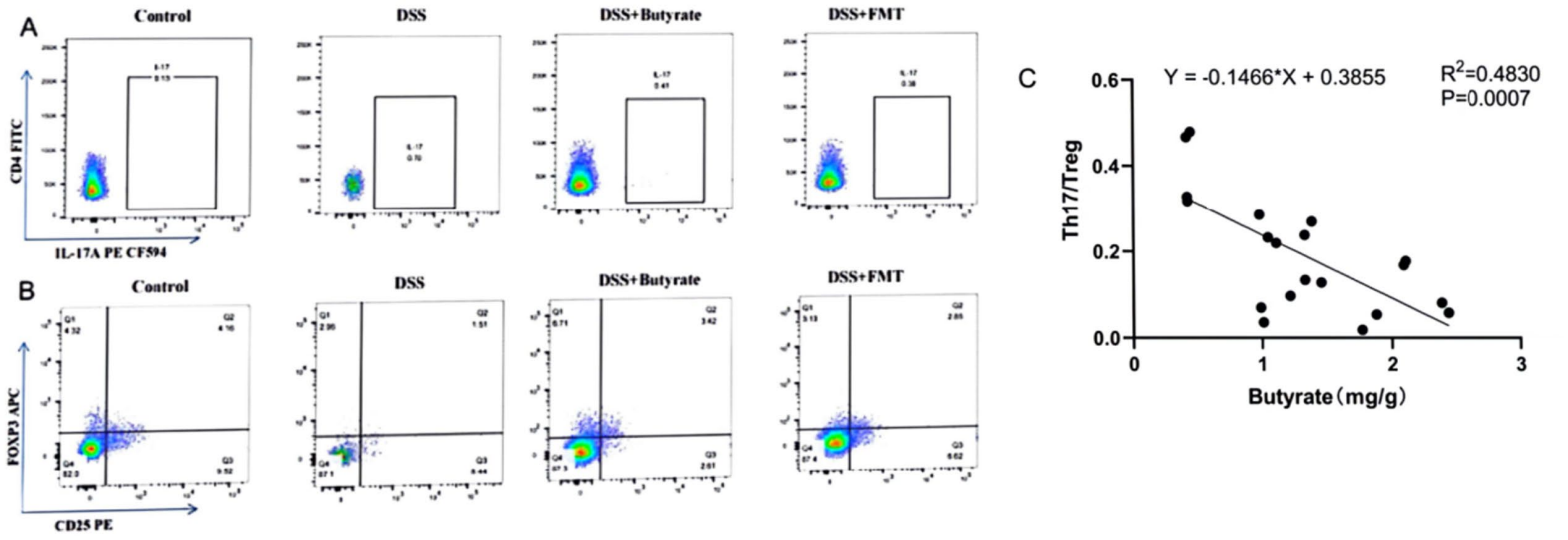
Flow cytometric analysis of Th17 and Treg cells in splenic single-cell suspensions. **(A)** Percentage of CD4^+^IL-17A^+^ T cells in CD4^+^ T cell populations; **(B)** percentage of CD4^+^CD25^+^FoxP3^+^ Treg cells in CD4^+^ T cell populations (left to right: Control, DSS group, DSS + Butyrate group, DSS + FMT group); **(C)** correlation between fecal butyrate concentration and Th17/Treg ratio.

As illustrated in Figure 9 and Table 3, compared to the control group, DSS-treated mice exhibited a significant increase in Th17 cell proportion (*p* < 0.01), a marked reduction in Treg cells (*p* < 0.01), and a pronounced elevation in the Th17/Treg ratio (*p* < 0.001). In the butyrate-treated group, Th17 cell frequency decreased (*p* < 0.05), Treg proportion increased (*p* < 0.05), and the Th17/Treg ratio declined significantly (*p* < 0.001) relative to the DSS group. Similarly, the FMT group showed a substantial reduction in Th17 cells (*p* < 0.01) and Th17/Treg ratio (*p* < 0.01) compared to the DSS group. To evaluate the relationship between butyrate and Th17/Treg balance, correlation analysis revealed a negative association between intestinal butyrate concentration and Th17/Treg ratio (*p* = 0.0007) (Table 5).

**Table 5:** Th17 and Treg cell frequencies with Th17/Treg ratios across experimental groups.

Group	Th17 (%)	Treg (%)	Th17/Treg
Control	0.25 ± 0.13	4.06 ± 1.28	0.07 ± 0.05
DSS group	0.70 ± 0.34^**^	1.77 ± 0.51^**^	0.40 ± 0.13^***^
DSS + Butyrate group	0.37 ± 0.09^#^	3.00 ± 1.30	0.14 ± 0.07^###^
DSS + FMT group	0.35 ± 0.17^#^	2.03 ± 1.14**	0.20 ± 0.08^## *^

^*^P < 0.05 vs. Control; ^**^p < 0.01 vs. Control; ^***^p < 0.001 vs. Control; ^#^*p* < 0.05 vs. DSS group; ^##^*p* < 0.01 vs. DSS group; ^###^*p* < 0.001 vs. DSS group.

### 3.3 Comparative roles of butyrate and gut microbiota in anti-inflammatory processes

#### 3.3.1 Effects of FMT and butyrate interventions on general health and body weight

**Figure 10:**
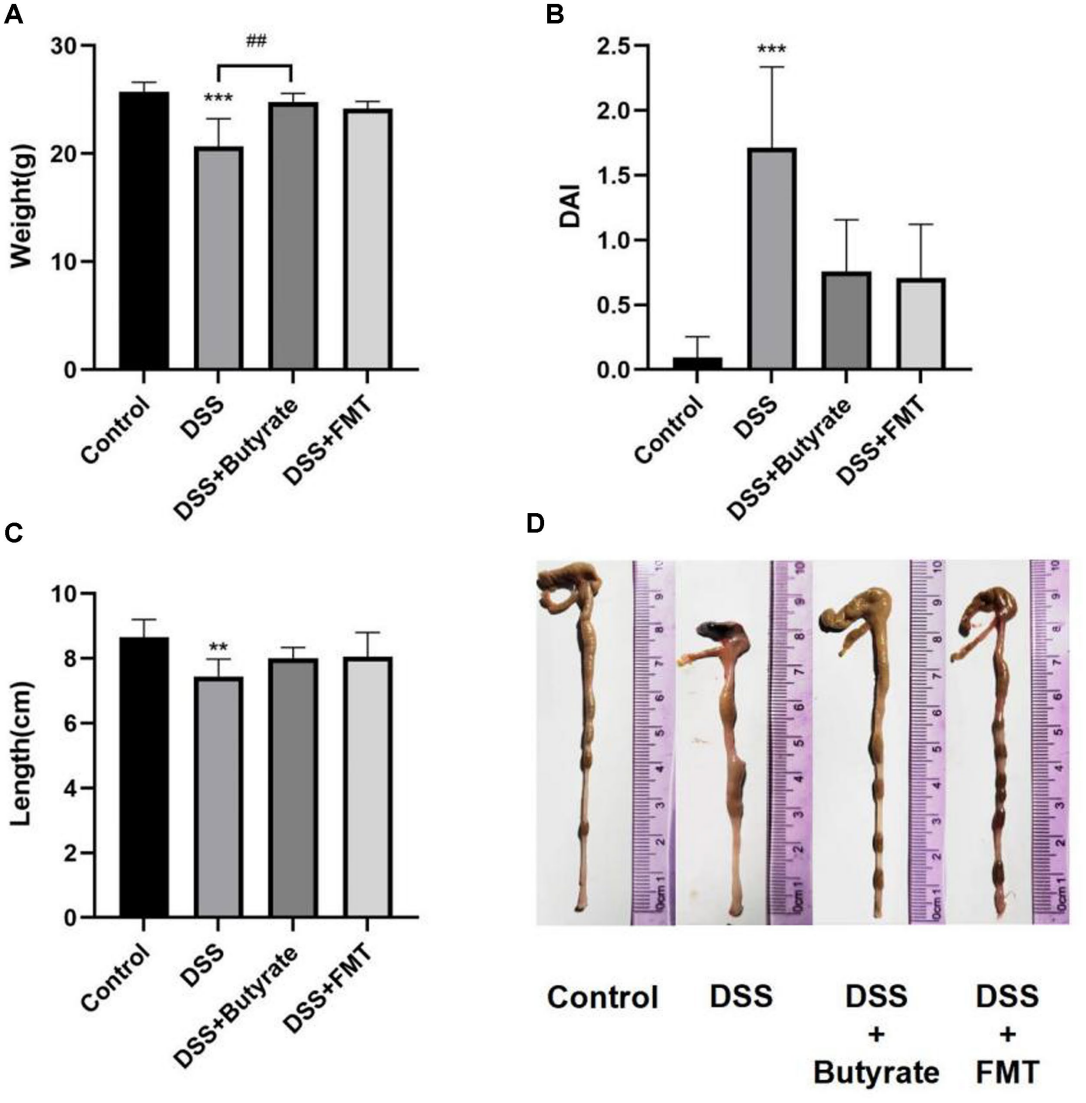
Butyrate and FMT alleviate weight loss and ameliorate colonic inflammation in UC mice. **(A)** Body weight at Day 14; **(B)** Disease Activity Index (DAI) scores; (C, D) Colon length measurements. ^*^p < 0.05, ^**^p < 0.01;^***^p < 0.001 vs. Control group; ^#^*p* < 0.05, ^##^*p* < 0.01, ^###^*p* < 0.001 vs. DSS group.

Compared to the control group, DSS-treated mice exhibited significant weight loss (*p* < 0.001). Butyrate supplementation markedly attenuated this weight reduction (*p* < 0.01 vs. DSS group), whereas FMT showed a non-significant trend toward weight recovery (Figure 10A). The DSS group also demonstrated a significantly elevated Disease Activity Index (DAI) score compared to controls (*p* < 0.001). Both FMT and butyrate treatments reduced DAI scores, though these values remained statistically comparable to the control group (*p* > 0.05) (Figure 10B). Macroscopically, DSS mice displayed severe colonic ulceration, mucosal hyperemia, edema, and shortened colon length (*p* < 0.01 vs. control). Butyrate and FMT interventions restored colon length to near-normal levels (Figures 10C,D), with minimal ulceration and only mild mucosal erosion or edema.

#### 3.3.2 Effects of FMT and butyrate on histopathology and HE staining

**Figure 11:**
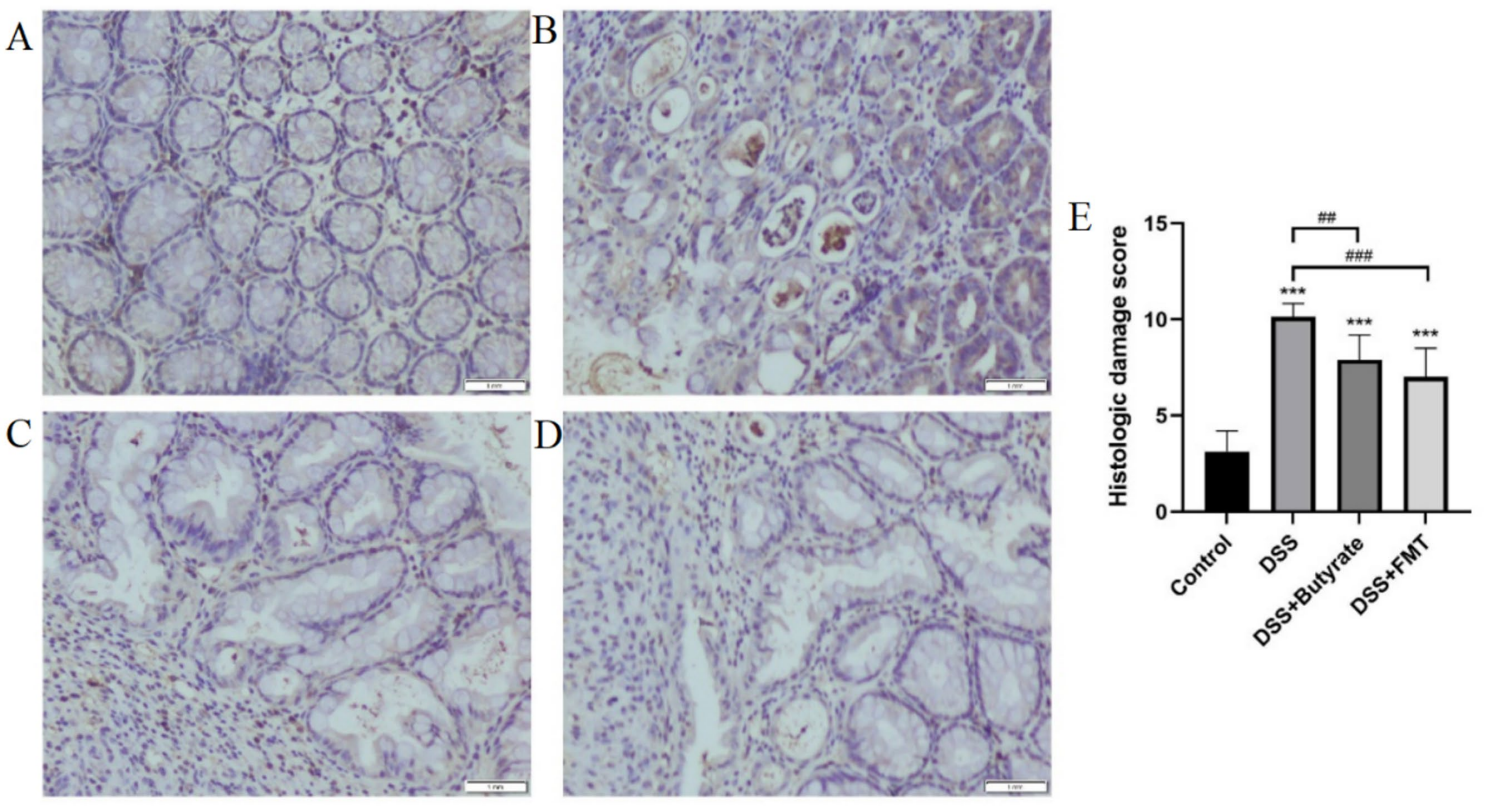
Histopathological evaluation of colonic tissues (H&E staining). **(A)** control group; **(B)** DSS group; **(C)** DSS + Butyrate group; **(D)** DSS + FMT group; **(E)** histopathological scoring bar plot. ^*^p < 0.001 vs. Control; ^##^*p* < 0.01 vs. DSS group; ^###^*p* < 0.001 vs. DSS group.

Control mice exhibited intact mucosal architecture with smooth surfaces. In contrast, DSS-treated mice showed disrupted glandular structures, severe neutrophilic infiltration, goblet cell depletion, and lamina propria vascular proliferation, resulting in significantly higher histopathological scores (*p* < 0.01 vs. control). Both butyrate and FMT interventions ameliorated these pathological changes, significantly lowering histopathological scores (*p* < 0.05) (Figure 11).

#### 3.3.3 Effects of FMT and butyrate on immunohistochemical staining in colonic tissue

**Figure 12:**
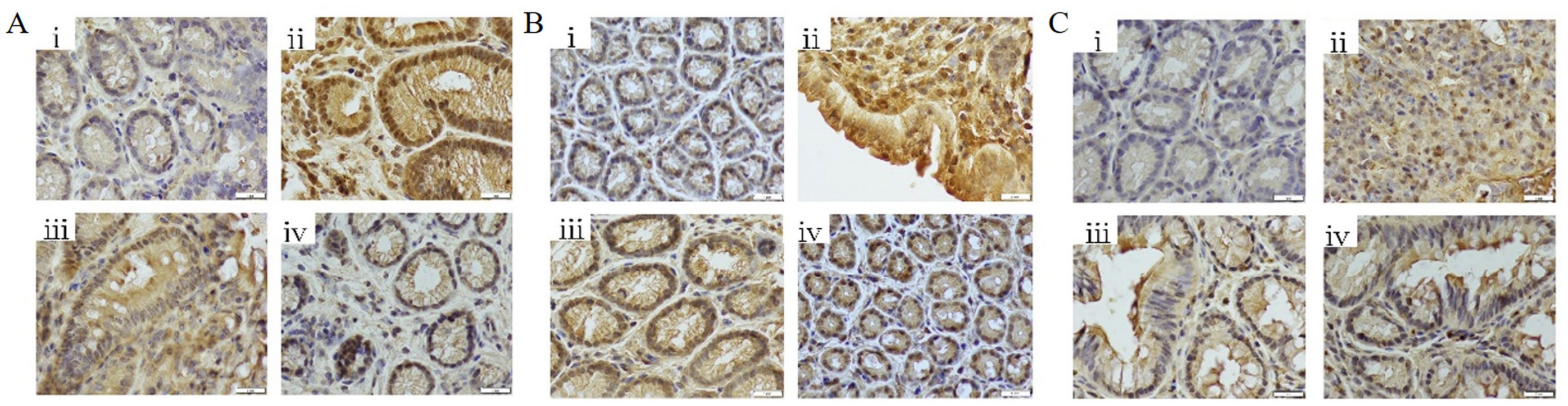
Immunohistochemical analysis of colonic tissues (400 × magnification). **(A)** NF-κB p65 expression levels; **(B)** STAT3 expression levels; **(C)** IL-6 expression levels. (i) Control; (ii) DSS group; (iii) DSS + Butyrate group; (iv) DSS + FMT group.

DSS treatment significantly upregulated the expression of NF-κB p65, STAT3, and IL-6 in colonic tissues compared to controls (*p* < 0.01). Both butyrate and FMT interventions suppressed the expression of these inflammatory markers (*p* < 0.05 vs. DSS group) (Figures 12A–C).

### 3.4 Synergistic effects of butyrate and gut microbiota

#### 3.4.1 Phylum-level composition of gut microbiota under FMT and butyrate interventions

**Figure 13:**
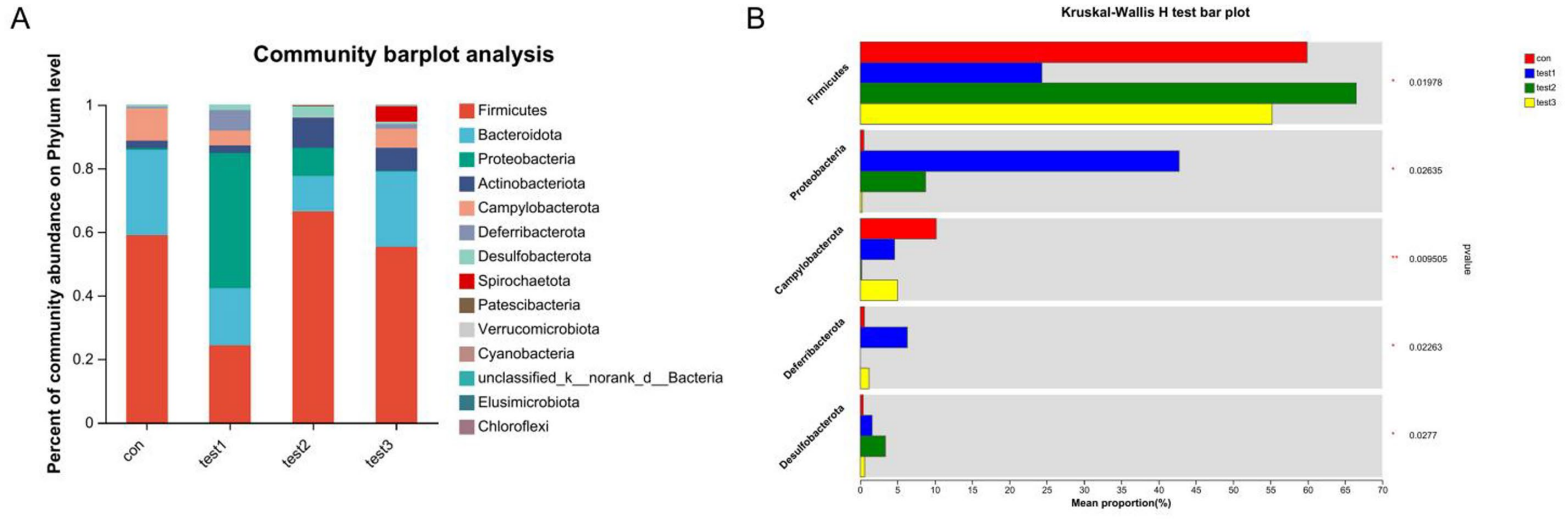
Phylum-level microbial composition analysis. **(A)** Bar plot of taxonomic composition (x-axis: groups; y-axis: relative abundance); **(B)** differential abundance testing across groups. Con: Control; Test1: DSS group; Test2: DSS + Butyrate group; Test3: DSS + FMT group. Significance: ^*^p < 0.05; ^**^p < 0.01; ^***^p < 0.001.

The dominant phyla across all four groups were Firmicutes, Bacteroidota, Proteobacteria, and Actinobacteriota. Intergroup comparisons revealed that butyrate-treated mice exhibited significantly increased abundances of Firmicutes and Desulfobacterota, alongside reduced proportions of Proteobacteria, Deferribacterota, and Campylobacterota compared to the DSS group (*p* < 0.05). Relative to the control group, butyrate intervention markedly decreased Campylobacterota while elevating Desulfobacterota and Proteobacteria (Figures 13A,B).

#### 3.4.2 Species-level composition of gut microbiota under FMT and butyrate interventions bacteroides

**Figure 14:**
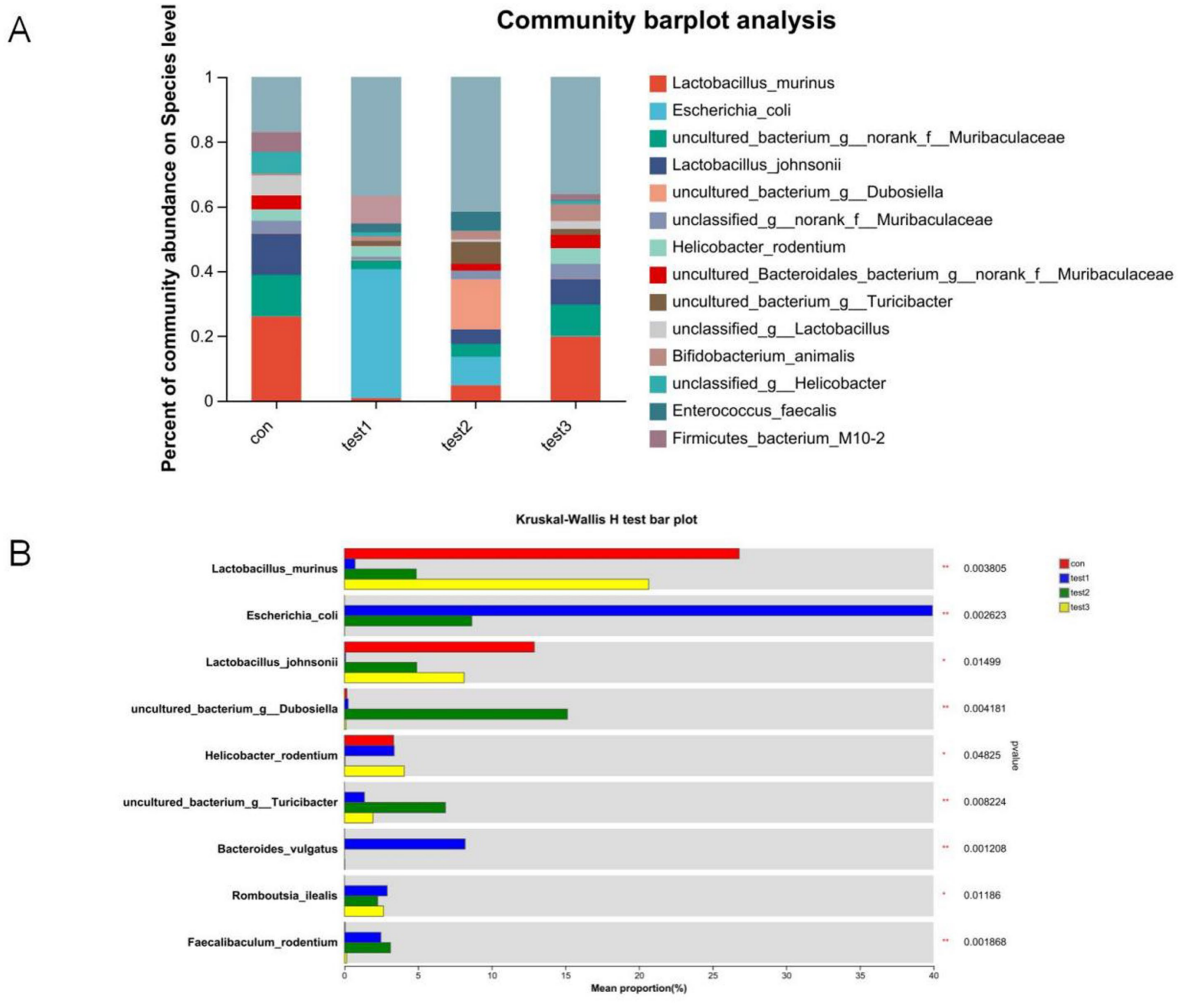
Comparative microbial community analysis. **(A)** Group-specific phylum abundance (x-axis: groups; y-axis: relative abundance); **(B)** Heatmap of differentially abundant taxa (y-axis: taxa; x-axis: mean relative abundance; rightmost column: *p*-values). Con: Control; Test1: DSS group; Test2: DSS + Butyrate group; Test3: DSS + FMT group. Significance: ^*^p < 0.05; ^**^p < 0.01; ^***^p < 0.001.

**Figure 15:**
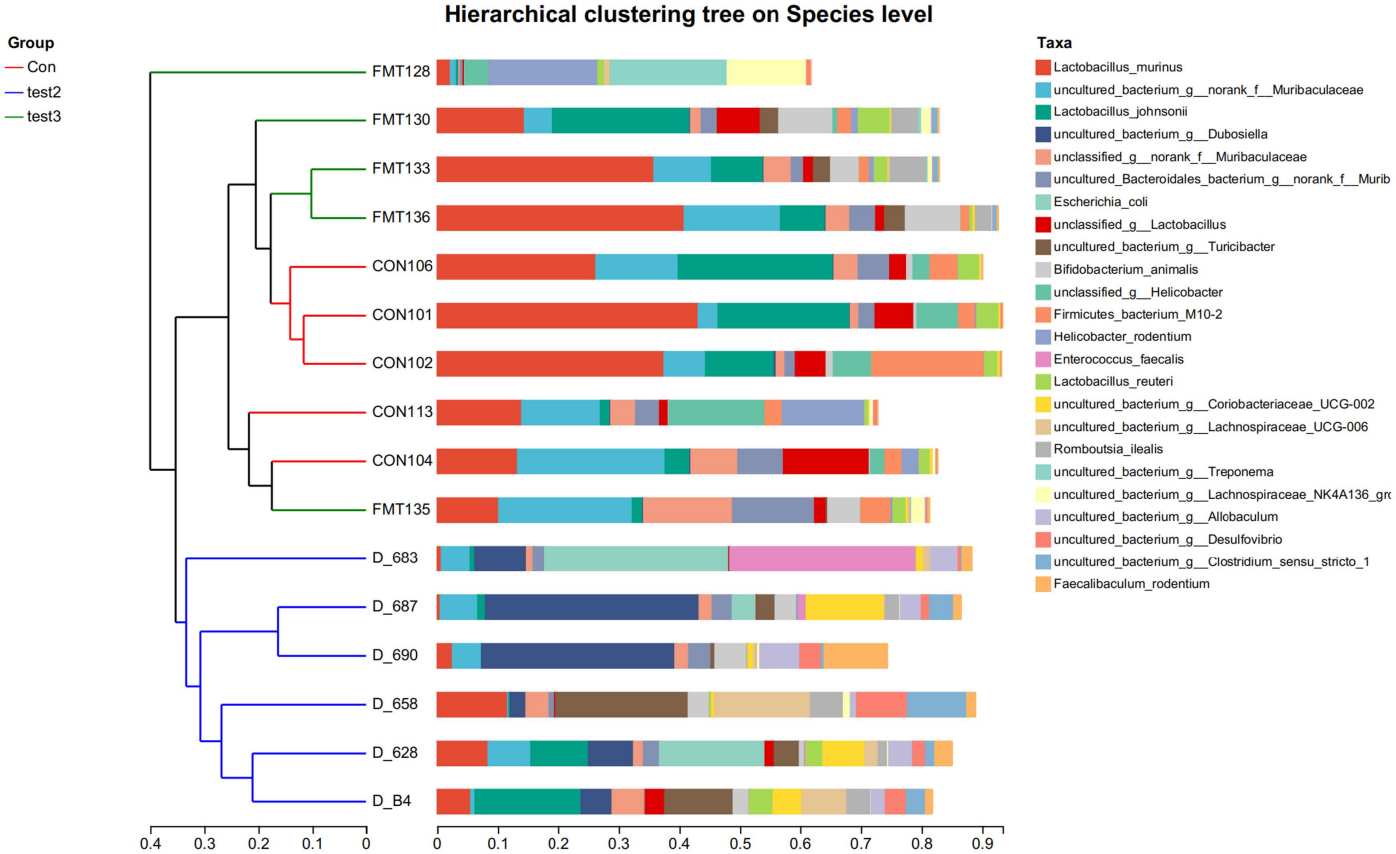
Hierarchical clustering tree on Species level Group-specific specie abundance (x-axis: relative abundance; y-axis:groups) Con: Control; Test2: Control + Butyrate group; Test3: Control + FMT group.

At the species level, the FMT-treated control mice demonstrated increased abundances of *Lactobacillus johnsonii* (potentially enhancing barrier function and immune regulation), *Muribaculaceae* (key mucin degraders and SCFA producers supporting gut homeostasis), and *Turicibacter* (implicated in bile acid metabolism and potentially anti-inflammatory), coupled with reduced proportions of *Escherichia coli* (often pro-inflammatory and barrier-disrupting, especially AIEC pathovars) and *Treponema* (associated with dysbiosis) compared to untreated controls. Similarly, FMT-treated DSS mice resulted in elevated levels of *Lactobacillus johnsonii*, *Muribaculaceae*, and *Turicibacter*, alongside decreased abundances of *Escherichia coli* and *Romboutsia ilealis* (a taxon frequently enriched in human IBD patients). Butyrate-treated control mice significantly increased the proportions of *Faecalibaculum rodentium* (a lactate producer potentially contributing to butyrate generation), *Dubosiella* (an acetate producer with anti-inflammatory potential), and *Turicibacter*, while diminishing *Helicobacter rodentium* (a potent murine pathogen known to drive colitis). Notably, Butyrate-treated DSS mice demonstrated a more pronounced shift, characterized by increased abundances of *Faecalibaculum rodentium*, *Dubosiella*, *Turicibacter*, and *Lactobacillus murinus* (another immunomodulatory Lactobacillus species), concurrent with significant reductions in *Escherichia coli* and *Helicobacter rodentium* (Figures 14A,B, 15). These collective shifts toward taxa associated with SCFA production, barrier enhancement, and anti-inflammatory effects, and away from known pathobionts and inflammation drivers, likely underpin the observed therapeutic benefits.

## 4 Discussion

### 4.1 Inflammatory and microbiota characteristics in acute vs. chronic UC

UC is an idiopathic, chronic inflammatory disease of the colonic mucosa, originating in the rectum and potentially extending to the entire colon (Ordás et al., 2012). Intestinal microbial dysbiosis plays a crucial role in UC pathogenesis (Chang, 2020). In this study, we successfully established acute and chronic UC mouse models using 3 and 2.5% DSS solutions, respectively. FMT was employed as an intervention to modulate gut microbiota, and colonic tissues were collected at the experimental endpoint. 16S rRNA amplicon sequencing revealed distinct microbiota profiles across groups. Acute UC mice exhibited reduced gut microbiota richness and diversity, whereas chronic UC mice showed only a moderate decline in diversity. Venn analysis demonstrated that operational taxonomic unit (OTU) composition in the acute UC group differed significantly from the control (CON) group, while the FMT group’s OTU profile closely resembled that of the CON group, indicating restored microbiota diversity and composition post-FMT (Figure 2B). Notably, the chronic UC group’s microbiota composition was closer to the CON group compared to the acute UC group (Figure 2C). *β-*diversity analysis via principal coordinates analysis (PCoA) and non-metric multidimensional scaling (NMDS) revealed distinct clustering among the CON, acute UC, and chronic UC groups, confirming marked differences in microbiota composition between UC models and controls. Post-FMT intervention, the FMT group clustered closer to the CON group, and chronic UC mice exhibited a tendency toward CON-like microbiota compared to acute UC mice (Figures 3D,E).

At the genus level, the acute UC group showed increased relative abundances of *Bacteroides* and *Escherichia-Shigella* but reduced *Ligilactobacillus*, *Lactobacillus*, and *Limosilactobacillus* compared to the CON group (Figures 4A–C; *p* < 0.05). In contrast, the chronic UC group displayed restored *Lactobacillus* levels without *Escherichia-Shigella* enrichment, alongside reduced *Dubosiella* abundance (Figures 4A,B,D). Species-level analysis revealed significant increases in *Lactobacillus johnsonii*, *Lactobacillus murinus*, and *Lactobacillus reuteri* in chronic UC mice, alongside decreased *Escherichia coli* and *Bacteroides vulgatus* (Figure 5D; *p* < 0.05). These findings suggest a “self-healing” tendency in gut microbiota during disease progression. Post-acute phase, mucosal repair and reduced inflammatory mediators create a microenvironment conducive to probiotic proliferation. Restoration of butyrate-producing bacteria further promotes microbiota equilibrium, highlighting the importance of timely probiotic interventions during disease remission.

### 4.2 Timing of FMT in acute vs. chronic UC

Most animal colitis models mimic acute UC, whereas clinical UC is characterized by alternating relapse and remission phases (Ordás et al., 2012). We established a chronic UC model using cyclical 2.5% DSS administration. Histopathological analysis revealed acute UC features (epithelial damage, mucosal edema, ulceration, goblet cell loss, and inflammatory infiltration), whereas chronic UC mice exhibited epithelial hyperplasia, fibrosis, lymphoid follicle hyperplasia, and milder inflammation. Both models showed reduced microbiota richness and diversity versus controls, though chronic UC mice exhibited higher *α*-diversity and closer OTU overlap with the CON group (PCoA and Venn analyses). Chronic UC mice also demonstrated restored Firmicutes, *Lactobacillus*, and *Ligilactobacillus* abundances without pathogenic *Escherichia-Shigella* enrichment (*p* < 0.05).

Flow cytometry revealed elevated Th17 cells, reduced Treg cells, and Th17/Treg imbalance in acute UC mice. In chronic UC, Treg proportions partially recovered, and Th17/Treg ratios decreased (*p* < 0.05). Th17 cells promote inflammation via IL-17, while Tregs maintain immune tolerance via TGF-β1 and IL-10 (Yamada et al., 2016; Noack and Miossec, 2014). These findings suggest gradual immune homeostasis restoration during chronic UC. Collectively, chronic UC mice exhibit microbiota and immune profiles closer to healthy controls, supporting optimal post-acute intervention timing for microbiota modulation.

### 4.3 Role and mechanisms of butyrate in UC anti-inflammation (balancing Th17/Treg)

Butyrate, a microbiota-derived metabolite with anti-inflammatory properties (Gao et al., 2023; Tye et al., 2018; Chang et al., 2014), ameliorates colitis by modulating Th17/Treg balance. Our data demonstrate that oral butyrate supplementation promotes butyrate-producing bacteria, reduces splenic Th17/Treg ratios, and alleviates UC. DSS-induced colitis models reliably replicate UC features without direct adaptive immune activation (Saleh and Elson, 2011). DSS-treated mice exhibited weight loss, bloody stools, elevated Th17, and reduced Tregs. Th17/Treg imbalance is central to UC immunopathology (Luo et al., 2017; Lee et al., 2018), and butyrate significantly reversed DSS-induced Th17/Treg elevation. Consistent with prior studies, butyrate promotes Treg differentiation while inhibiting Th17 polarization in IBD patients (Wen et al., 2021), restoring immune tolerance (Lv et al., 2018; Tong et al., 2021).

Butyrate’s anti-inflammatory mechanism involves suppression of the NF-κB/IL-6/STAT3 pathway. DSS activated NF-κB/IL-6/STAT3 signaling, which is initiated by damaged epithelial cells and innate immune cells (e.g., macrophages), releasing IL-6 to activate STAT3 in adaptive immune cells. Butyrate inhibited this cascade by stabilizing IκB*α*, blocking NF-κB nuclear translocation, and suppressing STAT3 activation (Kelly et al., 2004). IL-6 and STAT3 drive Th17 differentiation while inhibiting Tregs (Harbour et al., 2020; Durant et al., 2010; Bettelli et al., 2006), thereby skewing Th17/Treg balance and perpetuating inflammation (Liang et al., 2013; Zhao et al., 2021). Our findings suggest butyrate restores Th17/Treg balance via NF-κB/IL-6/STAT3 inhibition.

### 4.4 Comparative roles of butyrate vs. gut microbiota in anti-inflammation

Comparing DSS + FMT and DSS + butyrate groups, butyrate outperformed FMT in mitigating weight loss (*p* < 0.01; Figure 10A). Both interventions restored colon length (Figures 10C,D), improved histopathology (*p* < 0.05; Figure 11), and suppressed NF-κB p65, STAT3, and IL-6 expression (*p* < 0.05; Figure 12). Butyrate significantly reduced Th17/Treg ratios (*p* < 0.001) versus FMT (*p* < 0.01), with higher fecal butyrate levels. FMT restored microbiota to near-normal composition (Figures 3D,E).

Butyrate enriches beneficial taxa and suppresses pathogens, whereas FMT introduces donor-derived taxa. Dynamic interactions between butyrate, microbiota, and host immunity suggest synergistic therapeutic potential. Future studies should optimize combined regimens to enhance personalized UC management.

### 4.5 Synergy between butyrate and gut microbiota

UC pathogenesis involves reduced butyrate-producing bacteria (Machiels et al., 2014). Remarkably, butyrate supplementation increased *Lactobacillus johnsonii*, Faecalibaculum rodentium, Turicibacter, and Dubosiella—a probiotic correlating with elevated butyrate, anti-inflammatory IL-10, and reduced pro-inflammatory cytokines [Interleukin1β[IL-1β], IL-6, TNF-α] (He et al., 2019; Gilley et al., 2022; Ke et al., 2019; Cox et al., 2017; Wan et al., 2021). Butyrate supplementation enriches butyrate-producing taxa (e.g., Dubosiella) and suppresses pathobionts (e.g., Campylobacter), creating a self-reinforcing cycle: elevated butyrate further promotes symbiont growth while inhibiting pathogens via luminal acidification and redox modulation (Million and Raoult, 2018). This synergy aligns with clinical evidence showing FMT responders exhibit increased butyrogenic bacteria (e.g., *Lachnospiraceae*, *Roseburia*) and elevated fecal butyrate (Fuentes et al., 2017). Butyrate reciprocally enriches these commensals by providing preferred carbon sources via cross-feeding (Szabó et al., 2017) and Inhibiting pathogenic competitors (Enterobacteriaceae) through HDAC suppression (Byndloss et al., 2017). This bidirectional crosstalk amplifies anti-inflammatory effects on Treg/Th17 balance.

Furthermore, we observed a significant reduction of Firmicutes in the gut of UC mice. Previous studies have demonstrated that decreased abundance of Firmicutes in UC patients is associated with intestinal dysfunction and inflammatory responses (Ananthakrishnan, 2020). Our data revealed that butyrate supplementation reversed this reduction in Firmicutes and alleviated colonic inflammation. Another bacterium markedly reduced by butyrate was Campylobacter, which typically colonizes the human oral cavity. Gastrointestinal colonization of Campylobacter has been linked to intestinal inflammation in UC patients, and the pSma1 plasmid identified in Campylobacter is associated with severe UC (Liu et al., 2020; Gemmell et al., 2018). The ameliorative effects of butyrate on DSS-induced colitis may be partially attributed to its ability to reduce pathogenic bacterial abundance and enhance the relative abundance of novel butyrate-producing probiotics, including Dubosiella.

## 5 Conclusion

Restoration of intestinal homeostasis is widely recognized as pivotal in treating gut-related disorders. However, emerging laboratory evidence suggests that restoring gut health extends beyond merely reestablishing microbial abundance and composition. Critical considerations now include temporal compatibility of microbiota with host mucosal immunity, nutritional requirements of commensal bacteria, immune-modulatory effects of microbial metabolites, and the dynamic interplay between disease progression and host resistance. This necessitates adaptive therapeutic strategies rather than static interventions.

In this study, through comprehensive investigations of gut microbiota, butyrate metabolism, Th17/Treg immune regulation, and NF-κB/IL-6/STAT3 inflammatory signaling in murine models of acute and chronic IBD, we identified therapeutic advantages of microbiota restoration during disease remission—particularly when Th17/Treg ratios are optimally balanced—and highlighted the synergistic role of butyrate. These findings provide experimental foundations for personalized modulation of the gut ecosystem in IBD. Future studies will refine the timing of microbial interventions, optimize butyrate administration protocols, classify functionally relevant bacterial taxa, and delineate temporal relationships between epithelial pathology and therapeutic responses, aiming to yield clinically actionable insights.

## Data Availability

The raw sequences generated by 16S rRNA sequencing have been deposited in the NCBI Sequence Read Archive (PRJNA1298535; SRA: SRP604534).

## References

[ref1] Ananthakrishnan A. N. (2020). Microbiome-based biomarkers for IBD. Inflamm. Bowel Dis. 26, 1463–1469. doi: 10.1093/ibd/izaa071, PMID: 32311010

[ref2] Bettelli E. Carrier Y. Gao W. Korn T. Strom T. B. Oukka M.. (2006). Reciprocal developmental pathways for the generation of pathogenic effector TH17 and regulatory T cells. Nature 441, 235–238. doi: 10.1038/nature04753, PMID: 16648838

[ref3] Byndloss M. X. Olsan E. E. Rivera-Chávez F. Tiffany C. R. Cevallos S. A. Lokken K. L.. (2017). Microbiota-activated PPAR-γ signaling inhibits dysbiotic Enterobacteriaceae expansion. Science 357, 570–575. doi: 10.1126/science.aam9949, PMID: 28798125 PMC5642957

[ref4] Chang J. T. (2020). Pathophysiology of inflammatory bowel diseases. N. Engl. J. Med. 383, 2652–2664. doi: 10.1056/NEJMra2002697, PMID: 33382932

[ref5] Chang P. V. Hao L. Offermanns S. Medzhitov R. (2014). The microbial metabolite butyrate regulates intestinal macrophage function via histone deacetylase inhibition. Proc. Natl. Acad. Sci. USA 111, 2247–2252. doi: 10.1073/pnas.1322269111, PMID: 24390544 PMC3926023

[ref6] Cox L. M. Sohn J. Tyrrell K. L. Citron D. M. Lawson P. A. Patel N. B.. (2017). Description of two novel members of the family Erysipelotrichaceae: Ileibacterium valens gen. Nov., sp. nov. and Dubosiella newyorkensis, gen. Nov., sp. nov., from the murine intestine, and emendation to the description of Faecalibaculum rodentium. Int. J. Syst. Evol. Microbiol. 67, 1247–1254. doi: 10.1099/ijsem.0.001793, PMID: 28100298 PMC5817276

[ref7] Durant L. Watford W. T. Ramos H. L. Laurence A. Vahedi G. Wei L.. (2010). Diverse targets of the transcription factor STAT3 contribute to T cell pathogenicity and homeostasis. Immunity 32, 605–615. doi: 10.1016/j.immuni.2010.05.003, PMID: 20493732 PMC3148263

[ref8] Fuentes S. Rossen N. G. van der Spek M. J. Hartman J. H. Huuskonen L. Korpela K.. (2017). Microbial shifts and signatures of long-term remission in ulcerative colitis after faecal microbiota transplantation. ISME J. 11, 1877–1889. doi: 10.1038/ismej.2017.44, PMID: 28398347 PMC5520032

[ref9] Gao T. Feng M. Wang Z. Cao J. Chen Y. (2023). Microbiota-gut-adipose axis: butyrate-mediated the improvement effect on inflammatory response and fatty acid oxidation dysregulation attenuates obesity in sleep-restricted mice. Microbes Infect. 25:105125. doi: 10.1016/j.micinf.2023.105125, PMID: 36906253

[ref10] Gemmell M. R. Berry S. Mukhopadhya I. Hansen R. Nielsen H. L. Bajaj-Elliott M.. (2018). Comparative genomics of *Campylobacter concisus*: analysis of clinical strains reveals genome diversity and pathogenic potential. Emerg Microbes Infect 7:116. doi: 10.1038/s41426-018-0118-x, PMID: 29946138 PMC6018663

[ref11] Geremia A. Biancheri P. Allan P. Corazza G. R. Di Sabatino A. (2014). Innate and adaptive immunity in inflammatory bowel disease. Autoimmun. Rev. 13, 3–10. doi: 10.1016/j.autrev.2013.06.004, PMID: 23774107

[ref12] Gilley S. P. Ruebel M. L. Sims C. Zhong Y. Turner D. Lan R. S.. (2022). Associations between maternal obesity and offspring gut microbiome in the first year of life. Pediatr. Obes. 17:e12921. doi: 10.1111/ijpo.12921, PMID: 35478493 PMC9641193

[ref13] Gong Y. Lin Y. Zhao N. He X. Lu A. Wei W.. (2016). The Th17/Treg immune imbalance in ulcerative colitis disease in a Chinese Han population. Mediat. Inflamm. 2016:7089137. doi: 10.1155/2016/7089137 PMC4763012 26977120

[ref14] Harbour S. N. DiToro D. F. Witte S. J. Zindl C. L. Gao M. Schoeb T. R.. (2020). T(H)17 cells require ongoing classic IL-6 receptor signaling to retain transcriptional and functional identity. Sci Immunol 5:2262. doi: 10.1126/sciimmunol.aaw2262, PMID: 32680955 PMC7843024

[ref15] He T. Zhu Y. H. Yu J. Xia B. Liu X. Yang G. Y.. (2019). *Lactobacillus johnsonii* L531 reduces pathogen load and helps maintain short-chain fatty acid levels in the intestines of pigs challenged with *Salmonella enterica* Infantis. Vet. Microbiol. 230, 187–194. doi: 10.1016/j.vetmic.2019.02.003, PMID: 30827387

[ref16] Izcue A. Coombes J. L. Powrie F. (2009). Regulatory lymphocytes and intestinal inflammation. Annu. Rev. Immunol. 27, 313–338. doi: 10.1146/annurev.immunol.021908.132657, PMID: 19302043

[ref17] Ke X. Walker A. Haange S. B. Lagkouvardos I. Liu Y. Schmitt-Kopplin P.. (2019). Synbiotic-driven improvement of metabolic disturbances is associated with changes in the gut microbiome in diet-induced obese mice. Mol Metab 22, 96–109. doi: 10.1016/j.molmet.2019.01.012, PMID: 30792016 PMC6437638

[ref18] Kelly D. Campbell J. I. King T. P. Grant G. Jansson E. A. Coutts A. G.. (2004). Commensal anaerobic gut bacteria attenuate inflammation by regulating nuclear-cytoplasmic shuttling of PPAR-gamma and RelA. Nat. Immunol. 5, 104–112. doi: 10.1038/ni1018 14691478

[ref19] Lee S. H. Kwon J. E. Cho M. L. (2018). Immunological pathogenesis of inflammatory bowel disease. Intest Res 16, 26–42. doi: 10.5217/ir.2018.16.1.26, PMID: 29422795 PMC5797268

[ref20] Liang J. Nagahashi M. Kim E. Y. Harikumar K. B. Yamada A. Huang W. C.. (2013). Sphingosine-1-phosphate links persistent STAT3 activation, chronic intestinal inflammation, and development of colitis-associated cancer. Cancer Cell 23, 107–120. doi: 10.1016/j.ccr.2012.11.013, PMID: 23273921 PMC3578577

[ref21] Liu F. Chen S. Luu L. D. W. Lee S. A. Tay A. C. Y. Wu R.. (2020). Analysis of complete *Campylobacter concisus* genomes identifies genomospecies features, secretion systems and novel plasmids and their association with severe ulcerative colitis. Microb. Genom. 6:457. doi: 10.1099/mgen.0.000457, PMID: 33111662 PMC7725323

[ref22] Luo A. Leach S. T. Barres R. Hesson L. B. Grimm M. C. Simar D. (2017). The microbiota and epigenetic regulation of T helper 17/regulatory T cells: in search of a balanced immune system. Front. Immunol. 8:417. doi: 10.3389/fimmu.2017.00417, PMID: 28443096 PMC5385369

[ref23] Lv Q. Shi C. Qiao S. Cao N. Guan C. Dai Y.. (2018). Alpinetin exerts anti-colitis efficacy by activating AhR, regulating miR-302/DNMT-1/CREB signals, and therefore promoting Treg differentiation. Cell Death Dis. 9:890. doi: 10.1038/s41419-018-0814-4, PMID: 30166541 PMC6117360

[ref24] Machiels K. Joossens M. Sabino J. De Preter V. Arijs I. Eeckhaut V.. (2014). A decrease of the butyrate-producing species Roseburia hominis and *Faecalibacterium prausnitzii* defines dysbiosis in patients with ulcerative colitis. Gut 63, 1275–1283. doi: 10.1136/gutjnl-2013-304833, PMID: 24021287

[ref25] Million M. Raoult D. (2018). Linking gut redox to human microbiome. Hum. Microbiome J. 10, 27–32. doi: 10.1016/j.humic.2018.07.002

[ref26] Noack M. Miossec P. (2014). Th17 and regulatory T cell balance in autoimmune and inflammatory diseases. Autoimmun. Rev. 13, 668–677. doi: 10.1016/j.autrev.2013.12.004, PMID: 24418308

[ref27] Ohnmacht C. Park J. H. Cording S. Wing J. B. Atarashi K. Obata Y.. (2015). MUCOSAL IMMUNOLOGY. The microbiota regulates type 2 immunity through RORγt^+^ T cells. Science 349, 989–993. doi: 10.1126/science.aac4263, PMID: 26160380

[ref28] Ordás I. Eckmann L. Talamini M. Baumgart D. C. Sandborn W. J. (2012). Ulcerative colitis. Lancet 380, 1606–1619. doi: 10.1016/S0140-6736(12)60150-0, PMID: 22914296

[ref29] Paramsothy S. Kamm M. A. Kaakoush N. O. Walsh A. J. van den Bogaerde J. Samuel D.. (2017). Multidonor intensive faecal microbiota transplantation for active ulcerative colitis: a randomised placebo-controlled trial. Lancet 389, 1218–1228. doi: 10.1016/S0140-6736(17)30182-4, PMID: 28214091

[ref30] Saleh M. Elson C. O. (2011). Experimental inflammatory bowel disease: insights into the host-microbiota dialog. Immunity 34, 293–302. doi: 10.1016/j.immuni.2011.03.008, PMID: 21435584 PMC3108903

[ref31] Salvi P. S. Cowles R. A. (2021). Butyrate and the intestinal epithelium: modulation of proliferation and inflammation in homeostasis and disease. Cells 10:775. doi: 10.3390/cells10071775, PMID: 34359944 PMC8304699

[ref32] Smith P. M. Howitt M. R. Panikov N. Michaud M. Gallini C. A. Bohlooly-Y M.. (2013). The microbial metabolites, short-chain fatty acids, regulate colonic Treg cell homeostasis. Science 341, 569–573. doi: 10.1126/science.1241165, PMID: 23828891 PMC3807819

[ref33] Szabó G. Schulz F. Toenshoff E. Toenshoff E. R. Volland J. M. Finkel O. M.. (2017). Convergent patterns in the evolution of mealybug symbioses involving different intrabacterial symbionts. ISME J. 11, 715–726. doi: 10.1038/ismej.2016.148, PMID: 27983719 PMC5322300

[ref34] Tong L. Hao H. Zhang Z. Lv Y. Liang X. Liu Q.. (2021). Milk-derived extracellular vesicles alleviate ulcerative colitis by regulating the gut immunity and reshaping the gut microbiota. Theranostics 11, 8570–8586. doi: 10.7150/thno.62046, PMID: 34373759 PMC8344018

[ref35] Tye H. Yu C. H. Simms L. A. de Zoete M. R. Kim M. L. Zakrzewski M.. (2018). NLRP1 restricts butyrate producing commensals to exacerbate inflammatory bowel disease. Nat. Commun. 9:3728. doi: 10.1038/s41467-018-06125-0, PMID: 30214011 PMC6137172

[ref36] Ueno A. Jeffery L. Kobayashi T. Hibi T. Ghosh S. Jijon H. (2018). Th17 plasticity and its relevance to inflammatory bowel disease. J. Autoimmun. 87, 38–49. doi: 10.1016/j.jaut.2017.12.004, PMID: 29290521

[ref37] Voskens C. Stoica D. Rosenberg M. Vitali F. Zundler S. Ganslmayer M.. (2023). Autologous regulatory T-cell transfer in refractory ulcerative colitis with concomitant primary sclerosing cholangitis. Gut 72, 49–53. doi: 10.1136/gutjnl-2022-327075, PMID: 35428657 PMC9763232

[ref38] Wan F. Han H. Zhong R. Wang M. Tang S. Zhang S.. (2021). Dihydroquercetin supplement alleviates colonic inflammation potentially through improved gut microbiota community in mice. Food Funct. 12, 11420–11434. doi: 10.1039/D1FO01422F, PMID: 34673859

[ref39] Wang X. Sun Y. Zhao Y. Ding Y. Zhang X. Kong L.. (2016). Oroxyloside prevents dextran sulfate sodium-induced experimental colitis in mice by inhibiting NF-κB pathway through PPARγ activation. Biochem. Pharmacol. 106, 70–81. doi: 10.1016/j.bcp.2016.02.019, PMID: 26947454

[ref40] Wen S. He L. Zhong Z. Zhao R. Weng S. Mi H.. (2021). Stigmasterol restores the balance of Treg/Th17 cells by activating the butyrate-PPARγ axis in colitis. Front. Immunol. 12:741934. doi: 10.3389/fimmu.2021.741934, PMID: 34691046 PMC8526899

[ref41] Yamada A. Arakaki R. Saito M. Tsunematsu T. Kudo Y. Ishimaru N. (2016). Role of regulatory T cell in the pathogenesis of inflammatory bowel disease. World J. Gastroenterol. 22, 2195–2205. doi: 10.3748/wjg.v22.i7.2195, PMID: 26900284 PMC4734996

[ref42] Zhang M. Zhou L. Wang Y. Dorfman R. G. Tang D. Xu L.. (2019). *Faecalibacterium prausnitzii* produces butyrate to decrease c-Myc-related metabolism and Th17 differentiation by inhibiting histone deacetylase 3. Int. Immunol. 31, 499–514. doi: 10.1093/intimm/dxz022, PMID: 30809639

[ref43] Zhao Y. Luan H. Jiang H. Xu Y. Wu X. Zhang Y.. (2021). Gegen Qinlian decoction relieved DSS-induced ulcerative colitis in mice by modulating Th17/Treg cell homeostasis via suppressing IL-6/JAK2/STAT3 signaling. Phytomedicine 84:153519. doi: 10.1016/j.phymed.2021.153519, PMID: 33640781

